# C/EBPα-p30 confers AML cell susceptibility to the terminal unfolded protein response and resistance to Venetoclax by activating DDIT3 transcription

**DOI:** 10.1186/s13046-024-02975-3

**Published:** 2024-03-13

**Authors:** Mengbao Du, Mowang Wang, Meng Liu, Shan Fu, Yu Lin, Yankun Huo, Jian Yu, Xiaohong Yu, Chong Wang, Haowen Xiao, Limengmeng Wang

**Affiliations:** 1 Bone Marrow Transplantation Center of The First Affiliated Hospital & Liangzhu Laboratory, Zhejiang University School of Medicine, No.79 Qingchun Rd., Hangzhou, 310003 Zhejiang Province People’s Republic of China; 2 Department of Hematology, Sir Run Run Shaw Hospital, Zhejiang University School of Medicine, Hangzhou, China; 3 Institute of Hematology, Zhejiang University, Hangzhou, China; 4 Zhejiang Province Engineering Laboratory for Stem Cell and Immunity Therapy, Hangzhou, China; 5 Department of Hematological Malignancies Translational Science, Gehr Family Center for Leukemia Research, Hematologic Malignancies and Stem Cell Transplantation Institute, Beckman Research Institute, City of Hope Medical Center, Duarte, CA USA; 6 Hematology Department, The First Affiliated Hospital of Zhengzhou University, No. 1 Jianshe Dong Rd., Zhengzhou, 450000 Henan Province People’s Republic of China

**Keywords:** CCAAT-enhancer-binding protein α, *CEBPA* mutation, Acute myeloid leukemia, DNA damage-inducible transcript 3, Unfolded protein response, Endoplasmic reticulum stress, Venetoclax resistance

## Abstract

**Background:**

Acute myeloid leukemia (AML) with biallelic (*CEBPA*^bi^) as well as single mutations located in the bZIP region is associated with a favorable prognosis, but the underlying mechanisms are still unclear. Here, we propose that two isoforms of C/EBPα regulate DNA damage-inducible transcript 3 (DDIT3) transcription in AML cells corporately, leading to altered susceptibility to endoplasmic reticulum (ER) stress and related drugs.

**Methods:**

Human AML cell lines and murine myeloid precursor cell line 32Dcl3 cells were infected with recombinant lentiviruses to knock down *CEBPA* expression or over-express the two isoforms of C/EBPα. Quantitative real-time PCR and western immunoblotting were employed to determine gene expression levels. Cell apoptosis rates were assessed by flow cytometry. CFU assays were utilized to evaluate the differentiation potential of 32Dcl3 cells. Luciferase reporter analysis, ChIP-seq and ChIP-qPCR were used to validate the transcriptional regulatory ability and affinity of each C/EBPα isoform to specific sites at *DDIT3* promoter. Finally, an AML xenograft model was generated to evaluate the in vivo therapeutic effect of agents.

**Results:**

We found a negative correlation between *CEBPA* expression and DDIT3 levels in AML cells. After knockdown of *CEBPA*, DDIT3 expression was upregulated, resulting in increased apoptotic rate of AML cells induced by ER stress. *Cebpa* knockdown in mouse 32Dcl3 cells also led to impaired cell viability due to upregulation of Ddit3, thereby preventing leukemogenesis since their differentiation was blocked. Then we discovered that the two isoforms of C/EBPα regulate *DDIT3* transcription in the opposite way. C/EBPα-p30 upregulated *DDIT3* transcription when C/EBPα-p42 downregulated it instead. Both isoforms directly bound to the promoter region of *DDIT3.* However, C/EBPα-p30 has a unique binding site with stronger affinity than C/EBPα-p42. These findings indicated that balance of two isoforms of C/EBPα maintains protein homeostasis and surveil leukemia, and at least partially explained why AML cells with disrupted C/EBPα-p42 and/or overexpressed C/EBPα-p30 exhibit better response to chemotherapy stress. Additionally, we found that a low C/EBPα p42/p30 ratio induces resistance in AML cells to the BCL2 inhibitor venetoclax since BCL2 is a major target of DDIT3. This resistance can be overcome by combining ER stress inducers, such as tunicamycin and sorafenib in vitro and in vivo.

**Conclusion:**

Our results indicate that AML patients with a low C/EBPα p42/p30 ratio (e.g., *CEBPA*^bi^) may not benefit from monotherapy with BCL2 inhibitors. However, this issue can be resolved by combining ER stress inducers.

**Supplementary Information:**

The online version contains supplementary material available at 10.1186/s13046-024-02975-3.

## Background

Acute myeloid leukemia (AML) is a rapidly-progressing heterogeneous hematologic malignancy characterized by infiltration of the bone marrow, blood, and other tissues by myeloblasts or progranulocytes that fail to undergo normal differentiation [1, 2]. Over the past decade, advances in next-generation sequencing (NGS) have greatly improved our understanding of the molecular heterogeneity of AML [3]. Driven by these discoveries, improved disease classification and several new promising targeted therapies for AML have been developed [4, 5].

CCAAT-enhancer-binding protein α (C/EBPα) is the first characterized member of the C/EBP family transcription factors (TFs) and one of the most essential lineage-specific TFs involved in myeloid differentiation [6]. The mRNA encoding C/EBPα contains three AUGs that share a common open reading frame and produces two major protein isoforms via alternative translation initiation [7]. Translation from AUG1/2 produces full-length 42 kDa C/EBPα isoform p42 (CEBPα-p42), while translation from AUG3 produces the N-terminal truncated isoform p30 (C/EBPα-p30). The typical structure of C/EBPα consists of two main parts: transactivation domains (TADs) located at the N-terminal that are responsible for interactions with the transcription initiation complex and other proteins, and a basic region leucine zipper domain (bZIP) situated at the C-terminal in charge of binding to the specific DNA region [7]. C/EBPα-p30 lacks the first major TAD and inhibits the function of C/EBPα-p42 in a dominant-negative manner [8–10]. *CEBPA* mutations were found in approximately 10% of patients with AML [11]. N-terminal frameshift mutations usually lead to the termination of primary translation and shifting of translation to the downstream start codon, eventually generating an overexpressed C/EBPα-p30 isoform. C-terminal mutations are mostly insertion/deletion in-frame mutations, leading to impaired DNA-binding function [7]. Among AML patients with *CEBPA* mutations, 70% have both alleles affected, termed as *CEBPA* biallelically mutated (*CEBPA*^bi^) AML, where one allele usually carries an out-of-frame N-terminal mutation, while the other carries an in-frame C-terminal mutation [6, 12]. AML with biallelic (*CEBPA*^bi^) as well as single mutations located in the bZIP region has been identified to be associated with a favorable prognosis, but the mechanisms are still unclear [13, 14].

The unfolded protein response (UPR) is a conserved adaptive signal transduction pathway triggered by endoplasmic reticulum (ER) stress, which responds to the accumulation of unfolded or misfolded proteins in harsh physiological or pathological environments [15]. UPR first strives to restore protein homeostasis and maintain cell survival through a pro-survival arm called “adaptive UPR” [15]. However, in the setting of irremediable ER stress, the UPR transforms into pro-apoptotic “terminal UPR” signaling that promotes cell apoptosis [15, 16]. UPR signaling is reported to be important for the homeostasis of hematopoietic stem cells (HSCs) and their early transition to preleukemic stem cells (pre-LSCs) [17]. At the same time, it largely determines several biological properties of AML cells, including their ability to adapt and develop drug resistance, which are indicative of the clinical outcome of patients. AML-associated oncogenic proteins, such as MLL-AF6, MLL-AF9 and FLT3-ITD, can induce intrinsic ER stress in AML cells, resulting in higher sensitivity to the ER stress-inducing drug tunicamycin (TM) and the oxidative stress inducer arsenic trioxide (ATO) [18]. These studies strongly suggest the relationship between UPR signaling and AML-associated genetic abnormalities, as well as their potential in leukemia treatment.

DNA damage-inducible transcript 3 (DDIT3) is a key TF for terminal UPR as it regulates the expression of BCL2 family genes to induce apoptotic cell death [19]. DDIT3 is also known as C/EBP homologous protein (CHOP) and belongs to the C/EBP family. Several studies have revealed the mutual regulatory effects among members of this family. For instance, C/EBPγ acts as a trans-dominant negative inhibitor of C/EBPα and C/EBPβ [20, 21]. C/EBPα exerts its differentiation promoting function by interacting with the promoter of *CEBPG* and inhibiting its expression in AML [22]. This regulatory effect among members of C/EBP family inspired us to explore whether C/EBPα has a regulating effect on DDIT3, and the difference between two isoforms of C/EBPα as well as their effects on the response of AML cells to ER stress.

In the present study, we revealed that the two isoforms of C/EBPα exert antagonistic regulatory effects on DDIT3 expression. The C/EBPα-p30 isoform activates *DDIT3* transcription by binding to a unique site on its promoter. Meanwhile, C/EBPα-p42 suppresses *DDIT3* transcription, thereby impeding terminal UPR induction and helping normal myeloid progenitor cells to successfully complete the differentiation process. AML cells with suppressed C/EBPα-p42 and overexpressed C/EBPα-p30 exhibit increased susceptibility to apoptosis under ER stress. We further showed that a low p42/p30 ratio reduces the sensitivity of AML cells to the BCL2 inhibitor venetoclax, suggesting that AML patients with a low C/EBPα p42/p30 ratio (e.g., *CEBPA*^bi^) may not benefit from monotherapy using venetoclax. Moreover, we found that ER stress inducers tunicamycin and sorafenib could reverse venetoclax resistance through MCL1 inhibition and cell cycle regulation.

## Materials and methods

### AML patient samples

Bone marrow was aspirated from seven patients with de novo AML and seven healthy volunteers. The Ficoll density gradient was then used to recover viable mononuclear cells from the BM aspirates, and the cells were viably frozen and thawed for each experiment. The clinical characteristics of patients are described in Supplementary Table 1.

Approval for these studies was obtained from the ethics review committee of the First Affiliated Hospital, Zhejiang University School of Medicine (2020–385). Written informed consent was obtained from all the participants in accordance with the Declaration of Helsinki.

### Cell lines and cell culture

Human myeloid leukemia cell lines (Kasumi-1, K562, HEL, NB4, KG-1, MOLM-13, MV4-11, HL-60, and THP-1), 32Dcl3 murine myeloid cell line, and HEK293T cells were routinely cultured in our laboratory. Myeloid leukemia cells were maintained in RPMI-1640 medium (Corning, NY, USA) supplemented with 10% heat-inactivated fetal bovine serum (FBS; Gibco, Carlsbad, CA, USA). Murine 32Dcl3 cells were maintained in RPMI-1640 medium supplemented with 10% FBS and murine IL-3 (5 ng/ml; Peprotech, Rocky Hill, USA). HEK293T cells were maintained in Dulbecco's modified Eagle medium (DMEM; Corning) supplemented with 10% FBS.

### Plasmids and lentivirus infection

Human AML cell lines and 32Dcl3 murine myeloid cell line were transduced with recombinant lentiviruses and the plasmids used in this study have been previously described [23]. Detailed information is described in Supplemental Materials and Methods.

### Cell apoptosis assay

The Annexin V Apoptosis Detection Kit (Multi Sciences, Hangzhou, China) was used to detect apoptotic cell death according to the manufacturer’s instructions. Briefly, the cells were harvested and resuspended in 500 μL 1 × binding buffer with 5 μL APC-Annexin V and incubated for 5 min at room temperature in the dark. The cell apoptosis rate was determined as the percentage of Annexin V-positive cells by flow cytometry (Beckman Coulter, Inc., Miami, FL, USA), and data were processed using Flowjo V10.7.1. The drug-induced apoptosis rate was calculated as follows:

$$Drug\;induced\;apoptosis\%=Apoptosis\;by\;drug\%-Apoptosis\;by\;DMSO\%$$

### Cell cycle analysis

The cells (2 × 10^5^) were seeded in a 6-well plate and collected 24 h after incubation with the drugs or the corresponding volumes of DMSO. A Cell Cycle Staining Kit (Multi Sciences, Hangzhou, China) was used according to the manufacturer’s instructions for the detection of the living cell cycle. Analysis was conducted using flow cytometry (Beckman Coulter, Inc.), and the results were analyzed using FlowJo V10.7.1.

### Colony-forming unit (CFU) assays

Murine 32Dcl3 cells with *Cebpa* knockdown or scrambled control were plated in MethoCult™ GF M3434 medium (STEMCELL Technologies, Vancouver, Canada), which allows enumeration of BFU-E, CFU-GM, and CFU-GEMM based on morphology. After incubation for 12 days at 37°C and 5% CO_2_, colonies were photographed (× 40 magnification) using an inverted microscope, and manually counted, scored, and averaged.

### Quantitative real-time PCR

RNA analysis was extracted as previously described [23]. Specific procedures are described in Supplemental Materials and Methods. Sequences of the primers used are listed in Supplementary Table 2–3. The relative levels of mRNA in this study were analyzed using 2^−ΔΔCt^ method where Ct is the threshold cycle [24]. The Ct value of the target gene was normalized to that of *GAPDH/S16* (housekeeping genes) and the control (e.g., kasumi-1, scrambled, scrambled IL-3, and Vector). ΔΔCt was calculated as follows:

$$\mathrm{\Delta \Delta }{{\text{C}}}_{{\text{t}}}={{({\text{C}}}_{{\text{t}},Target }-{{\text{C}}}_{{\text{t}},Housekeeping})}_{Sample}-{{({\text{C}}}_{{\text{t}},Target }-{{\text{C}}}_{{\text{t}},Housekeeping})}_{Control}$$

### Western immunoblotting

Western immunoblotting analyses were carried out as previously described [23]. Detailed information is included in Supplemental Materials and Methods.

### Chromatin immunoprecipitation coupled with high-throughput sequencing (ChIP-seq) and ChIP-qPCR

The detailed information for ChIP-seq and ChIP-qPCR of HEK293T cells transfected with wild-type C/EBPα expression vector (p3 × FLAG-Myc-CMV-C/EBPα-p42) or empty vector was provided previously [23] and is included in Supplemental Materials and Methods. Sequences of primers used for ChIP-qPCR are listed in Supplementary Table 4.

### Luciferase reporter assays

Luciferase reporter assays were performed in HEK293T cells as described previously [23]. Specific procedures and construct information are included in Supplemental Materials and Methods.

### Leukemia xenograft assays

Male immune-deficient NOD/ShiLtJGpt-Prkdcem26/IL2rg em26/Gpt (NCG) mice (GemPharmatec, Jiangsu, China) were housed and bred at the Zhejiang Academy of Medical Sciences Central Animal Laboratory and used in the experiments at 8 weeks of age. Seven days after IV (tail vein) transplantation of 1 × 10^6^ luc/GFP-labeled NB4 cells, luminescence (AML burden) was quantified on treatment day 0 in each NCG mouse using bioluminescence imaging (BLI). Mice were then allocated to four treatment groups (Vehicle, Venetoclax, Sorafenib and Combo; five mice per group) so that each group had similar average luminescence on day 0. Then the mice in each group were orally administered the corresponding medication (by mouth; gavage). Luminescence of each mouse was assessed over time (day 7 and day 14) to reflect treatment response. The clinical behavior, appearance, body weight, and survival were also monitored.

The experiments were conducted in accordance with the protocols approved by The Experimental Animal Ethics Committee of the First Affiliated Hospital, Zhejiang University School of Medicine (2020–556).

### Statistical analysis

Data are expressed as mean ± SD of at least three independent experiments, and each group had three replicates. Different groups were compared using independent sample t-tests and Fisher’s exact tests. Linear regression analysis was used to determine the correlation between the expression of the two genes and other indicators. Statistical analyses were performed using the SPSS software (version 16.0). All probability values were generated using two-tailed tests. For in vivo experiments, the overall survival was depicted using a Kaplan–Meier curve, and the Log-rank test for trend was used to compare survival differences between the groups. *P* < 0.05 was considered as statistically significant, and *P* values between 0.05 and 0.1 were characterized as representing a trend. *P < 0.05; **P < 0.01, ***P < 0.001, and ****P < 0.0001.

## Results

### The endogenous expression of *CEBPA* in AML cells is negatively correlated with DDIT3 and cell susceptibility to terminal UPR

**Fig. 1:**
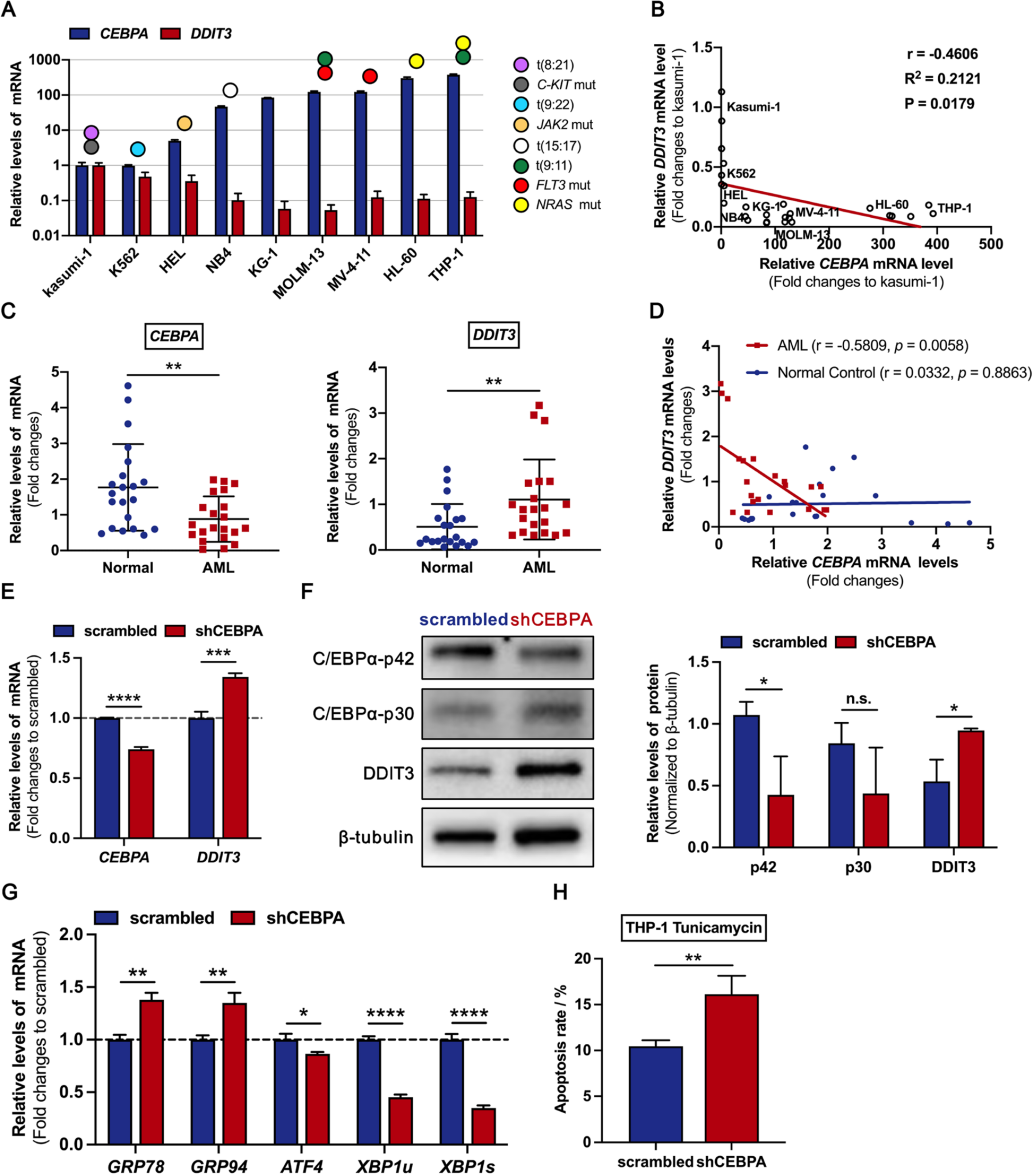
The endogenous expression of *CEBPA* in AML cells is negatively correlated with DDIT3 and cell susceptibility to terminal UPR. **A** Quantitative PCR analysis of endogenous expression of *CEBPA* and *DDIT3* in AML cell lines. **B** Correlation analysis using the Pearson model revealed a significant negative correlation between the mRNA levels of *CEBPA* and *DDIT3*. (C) Quantitative PCR analysis of endogenous *CEBPA* and *DDIT3* expression in cells from patients with de novo AML and healthy volunteers. Three technical replicates were performed for each sample. (D) The mRNA levels of *DDIT3* in primary AML cells were negatively correlated with *CEBPA*. **E**–**F** The *CEBPA* gene in THP-1 cells, with high endogenous C/EBPα expression and relatively low sensitivity to tunicamycin, was knocked down by shRNA lentiviral vector transfection. The down-regulated level of *CEBPA* and up-regulated level of DDIT3 were confirmed by quantitative PCR (**E**) and western immunoblotting analysis (**F**). **G** The mRNA levels of several genes encoding proteins in the pro-survival arm of the UPR were decreased after *CEBPA* knockdown in THP-1 cells. **H** Compared with the negative control group, THP-1 cells with *CEBPA* knockdown displayed a significantly increased apoptosis rate after the treatment with tunicamycin (100 ng/ml) for 48 h. Data represent Mean ± SD (*n* = 3); *P < 0.05; **P < 0.01, ***P < 0.001, ****P < 0.0001

In order to determine whether the expression of *DDIT3* is associated with *CEBPA* in AML, we first detected the endogenous expression of *DDIT3* and *CEBPA* in nine AML cell lines (Fig. 1A). Pearson’s correlation analysis revealed a significant negative correlation between the mRNA levels of *CEBPA* and *DDIT3* (r = -0.4606, *P* = 0.0179) (Fig. 1B). We then analyzed the gene expression profile in bone marrow mononuclear cells from patients with de novo AML and healthy volunteers. This analysis revealed that the mRNA level of *CEBPA* in AML cells from patients was significantly lower than that in healthy controls, as we previously reported [23], whereas the mRNA level of *DDIT3* was significantly higher in AML cells (Fig. 1C). Pearson’s correlation analysis confirmed a negative correlation between *CEBPA* and *DDIT3* expression in primary AML cells (r = -0.5809, *P* = 0.0058) (Fig. 1D). To further verify the negative correlation between *CEBPA* and *DDIT3* gene expression, the publicly available RNA-seq data from Beat AML project and microarray data from GEO (GSE38987) (https://www.ncbi.nlm.nih.gov/gds) of AML patients were analyzed, and negative linear correlation trends were also observed in these cases (Supplementary Fig. 1A-1B). We then induced ER stress in AML cell lines using tunicamycin, a canonical compound that disrupts protein maturation [25]. We found that cells with lower endogenous *CEBPA* expression, such as NB4, showed higher sensitivity to the terminal UPR, resulting in increased apoptosis (Supplementary Fig. 2A-2C). To further confirm the regulatory relationship between *CEBPA* and *DDIT3* expression, we knocked down *CEBPA* gene using shRNA in the THP-1 cell line, which has high endogenous C/EBPα expression (Fig. 1E-F). As expected, *DDIT3* expression was significantly upregulated after *CEBPA* knockdown (Fig. 1E-F). In addition, the spliced form of X-box binding protein 1 (XBP1s) levels in the pro-survival arm of the UPR were significantly reduced (Fig. 1G). These alterations preferentially lead the ER stress to terminal UPR manifested as mitochondria-dependent apoptotic pathway. Accordingly, the apoptosis rate of THP-1 cells under tunicamycin-induced ER stress increased after *CEBPA* knockdown (Fig. 1H and Supplementary Fig. 3). Taken together, these results suggest that C/EBPα is potentially involved in the regulation of expression of DDIT3 in AML cells, which in turn affects the sensitivity of AML cells to ER stress-induced cell death.

### *Cebpa* knockdown up-regulates Ddit3 expression and enhances myeloid progenitor cells susceptibility to ER stress mediated apoptosis

**Fig. 2:**
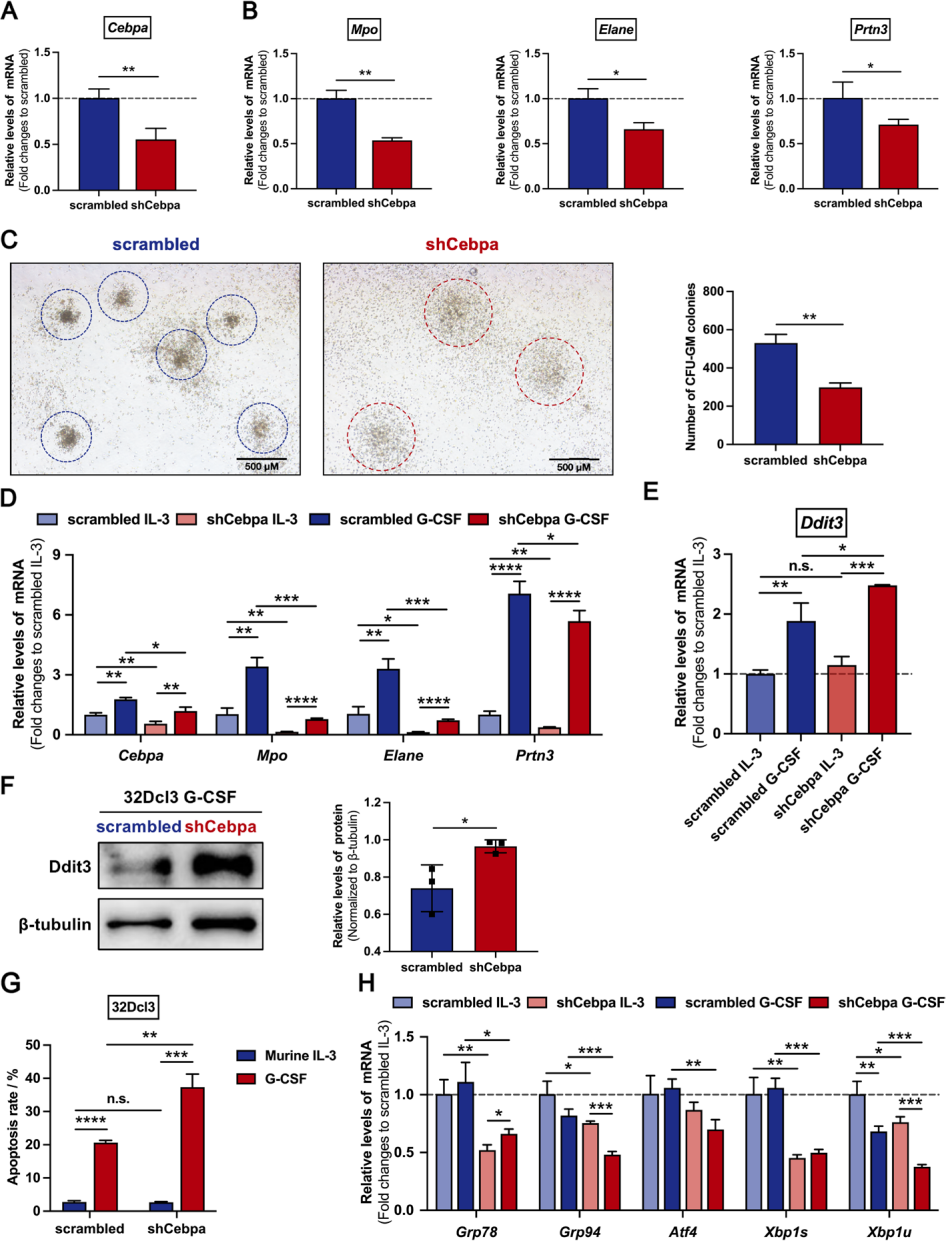
*Cebpa* knockdown up-regulates Ddit3 expression and enhances myeloid progenitor cells susceptibility to ER stress. **A** Quantitative PCR analysis revealed that the expression of *Cebpa* was downregulated in 32Dcl3-shCebpa cells compared with the negative control group. **B** Quantitative PCR analysis revealed that the expression of granulocytic differentiation markers *Mpo*, *Elane* and *Prtn3* was downregulated in 32Dcl3-shCebpa cells compared with the negative control group. **C** CFU assays revealed that *Cebpa* knockdown 32Dcl3 cells developed significantly fewer clones, and rare mature CFU-GM clones could be detected. × 40 magnification. Scale bar, 500 μm. **D** Quantitative PCR analysis revealed that the expression of *Cebpa* and granulocytic differentiation markers *Mpo*, *Elane* and *Prtn3* was continuously inhibited in 32Dcl3-shCebpa cells. **E**–**F** 32Dcl3 cells with *Cebpa* knockdown exhibit elevated mRNA (**E**) and protein (**F**) levels of *Ddit3* after granulocytic differentiation induced by G-CSF. **G** 32Dcl3 cells with *Cebpa* knockdown showed increased apoptotic rate than control cells during granulocytic differentiation induced by G-CSF (100 ng/ml, 48 h). **H** Quantitative PCR revealed that the mRNA levels of several genes encoding proteins in the pro-survival arm of the UPR were significantly decreased after *Cebpa* knockdown in 32Dcl3 cells. Data represent Mean ± SD (*n* = 3); *P < 0.05; **P < 0.01, ***P < 0.001, ****P < 0.0001

According to the pivotal role of *Cebpa* in granulocytic differentiation, we knocked down *Cebpa* in 32Dcl3 myeloid progenitor cells to study whether the effect of *Cebpa* on inducing myeloid differentiation was associated with its role in regulating UPR. After *Cebpa* knockdown (Fig. 2A), the expression of granulocytic differentiation markers *Mpo*, *Elane* and *Prtn3* in 32Dcl3 cells was significantly inhibited (Fig. 2B). CFU assays after 12 days of culture in proliferative environment revealed that the total number of clones of 32Dcl3-shCebpa cells was significantly less than that of 32Dcl3-scrambled cells, and rare mature CFU-GM clones could be detected for 32Dcl3-shCebpa cells (Fig. 2C). After inducing the granulocytic differentiation of 32Dcl3 cells by changing IL-3 in the culture medium to G-CSF for 48 h, the expression of granulocytic differentiation markers *Mpo*, *Elane* and *Prtn3* was upregulated in 32Dcl3-scrambled cells but still somewhat inhibited in 32Dcl3-shCebpa cells (Fig. 2D). Upregulated *Ddit3* expression accompanied by an increased rate of apoptosis was observed in 32Dcl3-shCebpa cells compared with 32Dcl3-scrambled cells after inducing granulocytic differentiation (Fig. 2E-G and Supplementary Fig. 4). In addition, several pro-survival UPR-related genes, including *Grp78*, *Grp94*, *Xbp1u*, and *Xbp1s*, were inhibited in 32Dcl3-shCebpa cells during granulocytic differentiation (Fig. 2H). We did not find an increase in the expression of *Atf4*, a positive regulator of *Ddit3*, indicating that *Atf4* was not responsible for the activation of *Ddit3* in this process (Fig. 2H). Generally, knockdown of *Cebpa* impairs the granulocytic differentiation potential of 32Dcl3 cells and makes them susceptible to spontaneous apoptosis by increasing the expression of *Ddit3*, eventually avoiding leukemogenesis.

### Overexpression of C/EBPα-p42 and C/EBPα-p30 in AML cells have different effects on regulating the UPR gene expression and the cell susceptibility to ER stress mediated apoptosis

**Fig. 3:**
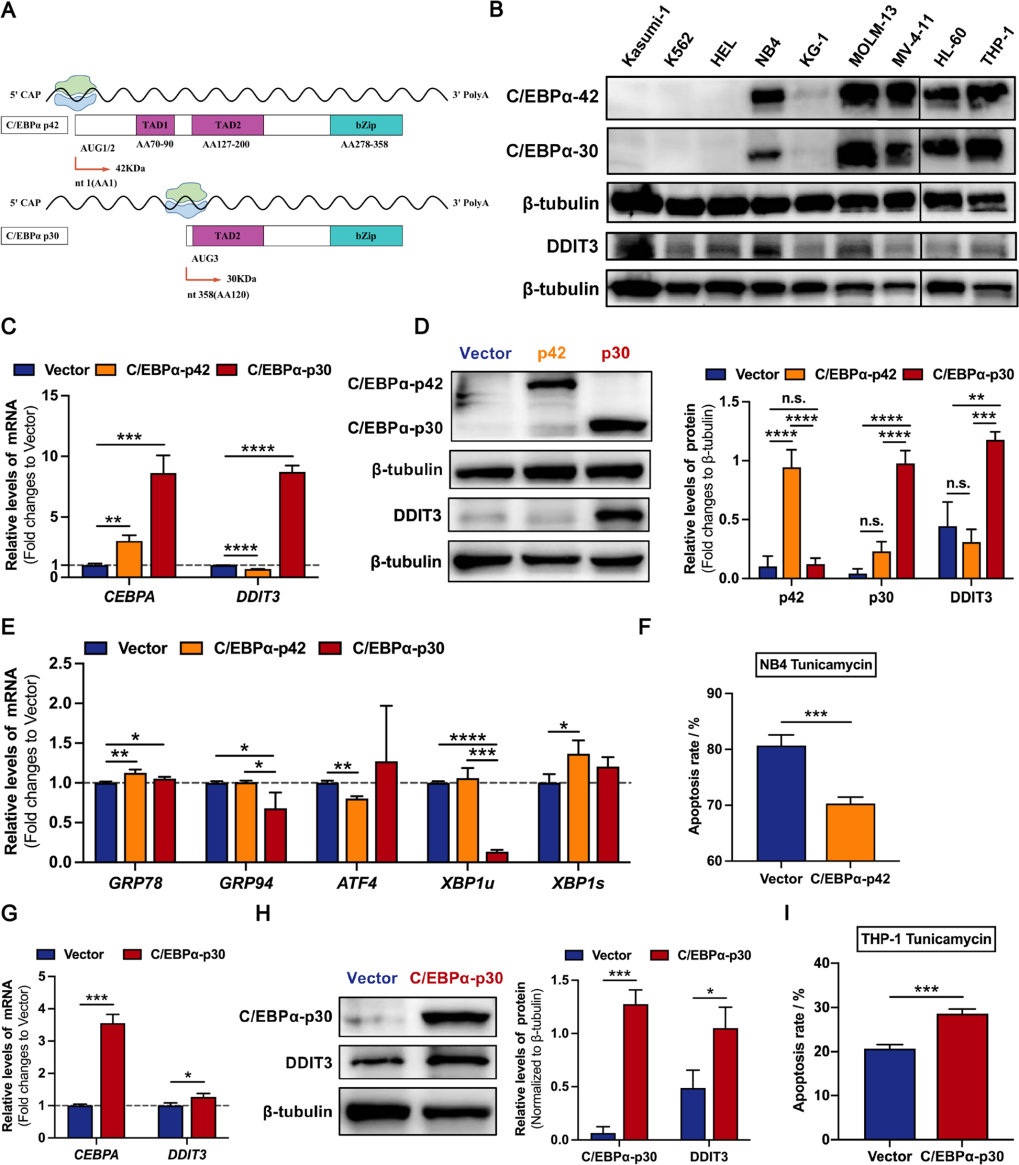
Overexpression of C/EBPα-p42 and C/EBPα-p30 in AML cells differently regulates the UPR genes and cell susceptibility to ER stress mediated apoptosis. **A** The pattern diagram of C/EBPα-p42 and C/EBPα-p30 protein translations. **B** Western immunoblotting analysis of endogenous expression of C/EBPα two isoforms and DDIT3 in AML cell lines. **C**-**D** The C/EBPα protein level in NB4 cells, with low endogenous C/EBPα expression and relatively high sensitivity to tunicamycin, was induced by the expression vectors of C/EBPα-p42 (pHIV7/SFFV-GFP-C/EBPα-p42) and C/EBPα-p30 (pHIV7/SFFV-GFP-C/EBPα-p30). The up-regulated levels of C/EBPα, and the correspondingly altered DDIT3 expression were confirmed by quantitative PCR (**C**) and western immunoblotting analysis (**D**). **E** Quantitative PCR revealed that the expression levels of the pro-survival genes *GRP94* and *XBP1u* were significantly decreased in NB4 cells with induction of C/EBPα-p30. **F** Compared with the vector control, the apoptosis rate of NB4 cells overexpressing C/EBPα-p42 was significantly reduced after the treatment with tunicamycin (100 ng/ml) for 48 h. **G**-**H** The C/EBPα protein level in THP-1 cells was induced by the expression vector of C/EBPα-p30 (pHIV7/SFFV-GFP-C/EBPα-p30). The up-regulated levels of C/EBPα-p30, and the correspondingly altered DDIT3 expression were confirmed by quantitative PCR (**G**) and western immunoblotting analysis (**H**). **I** Compared with the vector control, the apoptosis rate of THP-1 cells overexpressing C/EBPα-p30 was significantly increased after the treatment with tunicamycin (100 ng/ml) for 48 h. Data represent Mean ± SD (*n* = 3); *P < 0.05; **P < 0.01, ***P < 0.001, ****P < 0.0001

Full length C/EBPα protein is composed of the two TAD domains at the N-terminal and the bZIP domain at the C-terminal (Fig. 3A). Specially, C/EBPα-p30 lacks the first major TAD and shares a common bZIP compared to C/EBPα-p42 [26]. Increasing evidence has suggested that the regulatory effects of C/EBPα-p42 and C/EBPα-p30 on several genes are different or even opposite [27–29]. Correspondingly, a negative correlation trend was observed in AML cell lines between the C/EBPα p42/p30 ratio and DDIT3 at the protein level (Fig. 3B and Supplementary Fig. 5), which implied different functions of these two isoforms. To investigate the respective function of the two isoforms of C/EBPα in the regulation of DDIT3 expression, we overexpressed the full-length protein C/EBPα-p42 (pHIV7/SFFV-GFP-C/EBPα-p42) and truncated protein C/EBPα-p30 (pHIV7/SFFV-GFP-C/EBPα-p30) in NB4 cells (Fig. 3C-3D), which have relatively low levels of endogenous C/EBPα. The expression of DDIT3, both at the mRNA and protein levels, decreased after C/EBPα-p42 overexpression and increased after C/EBPα-p30 overexpression in NB4 cells (Fig. 3C-3D). In addition, the expression levels of several pro-survival genes in the UPR were significantly promoted by C/EBPα-p42, including *GRP78* and *XBP1s* (Fig. 3E). In contrast, after overexpression of C/EBPα-p30, the expression levels of the pro-survival genes *GRP94* and *XBP1u* significantly reduced in NB4 cells (Fig. 3E). We then detected the effect of C/EBPα-p42 on the sensitivity of NB4 cells to tunicamycin, which showed a high basal sensitivity to tunicamycin. After treatment with tunicamycin for 48 h, the apoptosis rate of NB4 cells overexpressing C/EBPα-p42 was significantly reduced compared to that of the vector control (Fig. 3F and Supplementary Fig. 6A). Due to the high sensitivity of NB4 cells to tunicamycin, C/EBPα-p30 overexpression is difficult to further increase the sensitivity of NB4 cells to tunicamycin. In that case, we chose to overexpress C/EBPα-p30 in THP-1 cells and repeat the above experiments. As a result, compared with the control group, DDIT3 expression was upregulated (Fig. 3G-H) and accompanied by an increased rate of apoptosis caused by tunicamycin (Fig. 3I and Supplementary Fig. 6B). These experiments indicate that C/EBPα two isoforms jointly participate in and differentially regulate the expression of DDIT3 to determine divergent cell fates.

### Both C/EBPα-p42 and C/EBPα-p30 can directly bind to *DDIT3* promoter but regulate its transcription in an antagonistic manner

**Fig. 4:**
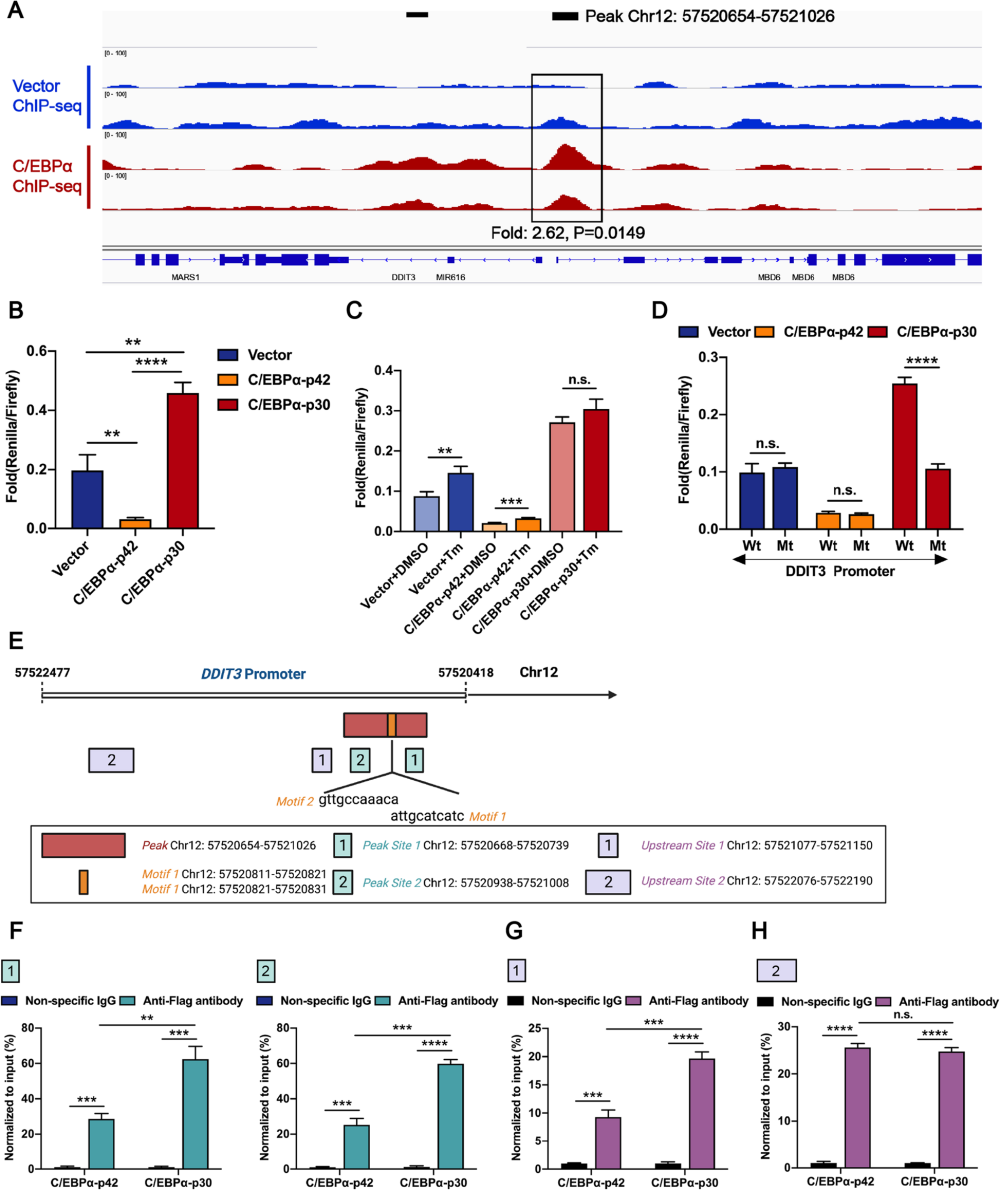
Both C/EBPα-p42 and C/EBPα-p30 directly bind to *DDIT3* promoter but regulate its transcription in an antagonistic manner. **A** ChIP-seq of C/EBPα revealed a C/EBPα binding site localized in the promoter region of *DDIT3* (Chr12:57520654–57521026). **B** C/EBPα-p42 inhibited the transcription of *DDIT3*, while C/EBPα-p30 exerted an activation effect. **C** After adding tunicamycin (100 ng/ml, 24 h) to induce the terminal UPR, the transcriptions of luciferase were further increased on the basis of the original lower level in the HEK293T cells expressing C/EBPα-p42 and C/EBPα-p30, and the cells in the control group. **D** When the ChIP-peak region (Chr12:57520654–57521026) was deleted, the activation effect of C/EBPα-p30 on *DDIT3* transcription was disrupted, whereas the suppression effect of C/EBPα-p42 was sustained. **E** Schematic diagram of two potential C/EBPα binding motifs determined by using the JASPAR database and the potential C/EBPα binding regions detected by ChIP-qPCR. **F**–**H** ChIP-qPCR revealed that C/EBPα-p30 showed stronger affinity than C/EBPα-p42 in (**F**) and near (**G**) the ChIP-peak region, while sharing the same affinity with C/EBPα-p42 at a position far from the peak (H). Data represent Mean ± SD (*n* = 3); *P < 0.05; **P < 0.01, ***P < 0.001, ****P < 0.0001

Since we observed that the expression and repression of DDIT3 were regulated by two C/EBPα isoforms, we questioned whether these regulations are mediated directly by C/EBPα-p42 and C/EBPα-p30. To identify potential C/EBPα-binding DNA sequences in the genome, we first expressed FLAG-tagged full-length C/EBPα in HEK293T cells and performed ChIP-seq. A peak of C/EBPα-binding DNA sequence (hg38, Chr12: 57520654–57521026) was found, which is localized at the promoter region of *DDIT3* (Fig. 4A). We then cloned the promoter sequence of *DDIT3* gene into luciferase reporter plasmids and co-transfected with *CEBPA* expression plasmids in HEK293T cells. As expected, the luciferase assay revealed that C/EBPα-p42 inhibited the transcription of *DDIT3*, while C/EBPα-p30 exerted an activation effect (Fig. 4B). After inducing terminal UPR with tunicamycin, transcription of *DDIT3* increased in cells transfected with vector control, and was further activated in cells transfected with C/EBPα-p30, but showed only a limited increase in cells transfected with C/EBPα-p42 (Fig. 4C). To determine the specific binding site of the two isoforms of C/EBPα on the *DDIT3* promoter, we deleted individual fragments of the *DDIT3* promoter in the luciferase reporter. As shown in Fig. 4D, when the peak region found by ChIP-seq was deleted, the activation effect of C/EBPα-p30 on *DDIT3* transcription was disrupted, whereas the suppression effect of C/EBPα-p42 was sustained. As for other fragments including Chr12: 57521027–57521326, Chr12: 57521327–57521626, Chr12: 57521627–57522036 and Chr12: 57522037–57522236 (Supplementary Fig. 7A), deletions did not affect the function of both C/EBPα-p42 and C/EBPα-p30 (Supplementary Fig. 7B). To confirm the binding of C/EBPα to the candidate fragments in *DDIT3* promoter, we first predicted the potential C/EBPα binding motifs by using the JASPAR database, and obtained two motifs in the ChIP-peak region with close positions and one base overlap, Chr12: 57520811–57520821 (motif 1) and Chr12: 57520821–57520831 (motif 2) (Fig. 4E). Then, we performed ChIP-qPCR in HEK293T cells expressing Flag-C/EBPα-p42 and Flag-C/EBPα-p30. As shown in the schematic diagram in Fig. 4E, both C/EBPα-p42 and C/EBPα-p30 could bind to the *DDIT3* promoter in multiple regions (Fig. 4F-H). However, C/EBPα-p30 showed stronger affinity than C/EBPα-p42 in and near the ChIP-peak region (Fig. 4F-G), and shared the same affinity with C/EBPα-p42 at a position far from the peak (Fig. 4H). These results indicate that C/EBPα-p42 and C/EBPα-p30 directly bind to the *DDIT3* promoter and exert opposite effects on its expression. C/EBPα-p30 has a special binding site in the proximal promoter region with high affinity to exert its activation function (Supplementary Fig. 7C).

### Reduction of C/EBPα p42/p30 ratio suppresses BCL2 and induces Venetoclax resistance by up-regulating DDIT3

**Fig. 5:**
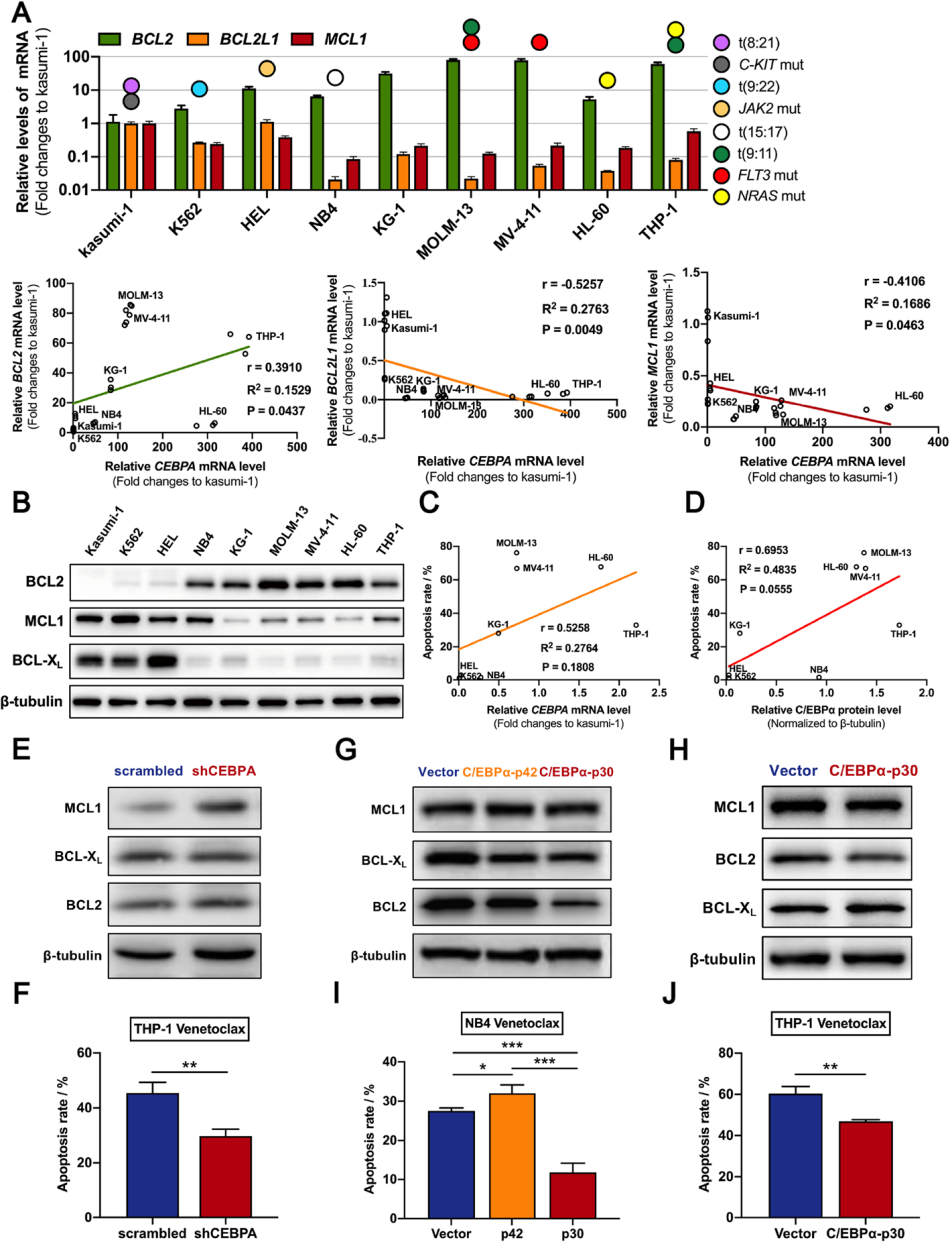
Reduction of C/EBPα p42/p30 ratio suppresses BCL2 and induces Venetoclax resistance by up-regulating DDIT3. **A** Quantitative PCR revealed that the mRNA level of *CEBPA* in AML cell lines was positively correlated with *BCL2*, but negatively correlated with *BCL2L1* and *MCL1*. **B** The protein levels of BCL2, BCL-X_L_, and MCL1 in AML cell lines by western immunoblotting analysis. **C**-**D** The sensitivity to venetoclax cytotoxicity was positively relevant to the mRNA (**C**) and protein (**D**) levels of *CEBPA* in AML cell lines. **E** The protein level of MCL1 was significantly increased in THP-1 cells with *CEBPA* knockdown. **F** Compared with the negative control group, THP-1 cells with *CEBPA* knockdown showed significantly decreased apoptosis rate induced by venetoclax (5 μM, 48 h). **G**-**H** The protein levels of BCL2 were significantly reduced in NB4 cells (**G**) and THP-1 cells (**H**) with induction of C/EBPα-p30. **I** Compared with the negative control group, NB4 cells with induction of C/EBPα-p42 showed significantly increased apoptosis rate induced by venetoclax (5 μM, 48 h), while cells with induction of C/EBPα-p30 showed significantly decreased apoptosis rate. **J** Compared with the negative control group, THP-1 cells with induction of C/EBPα-p30 showed significantly decreased apoptosis rate induced by venetoclax (5 μM, 48 h). Data represent Mean ± SD (*n* = 3); *P < 0.05; **P < 0.01, ***P < 0.001

The primary mechanism by which DDIT3 induces cell apoptosis is by suppressing the transcription of the anti-apoptotic protein BCL2 [16]. To investigate whether the regulation of DDIT3 directed by C/EBPα further affects the intrinsic apoptotic pathway, we analyzed the relationship between *CEBPA* and *BCL2* family proteins in nine AML cell lines (Fig. 5A). The mRNA level of *CEBPA* was positively correlated with *BCL2* (r = 0.3910, *P* = 0.0437), but negatively correlated with BCL-X_L_ encoding gene *BCL2L1* (r = -0.5257, *P* = 0.0049) and *MCL1* (r = -0.4106, *P* = 0.0463) (Fig. 5A). We also verified these correlations by using the RNA-seq data from Beat AML project and microarray data from GEO (GSE38987) (Supplementary Fig. 8A-8B). Similar correlations were also detected at the protein level by western immunoblotting analysis (Fig. 5B and Supplementary Fig. 8C). Since the two isoforms of C/EBPα protein have been shown to have opposite functions for DDIT3 expression, we further correlated the C/EBPα p42/p30 ratio with the level of anti-apoptotic proteins, and the results were consistent with those obtained for the total C/EBPα protein level (Supplementary Fig. 8D). We then detected the sensitivity of AML cells to the BCL2 inhibitor venetoclax (Supplementary Fig. 9 and Supplementary Fig. 10A). After treatment with venetoclax, the rate of apoptosis of AML cell lines increased with endogenous *CEBPA* (Fig. 5C-D) and *BCL2* expression (Supplementary Fig. 10B). To directly explore the relationship between C/EBPα and anti-apoptotic proteins, we detected changes in protein levels of BCL2, BCL-X_L_ and MCL1 in THP-1 cells after *CEBPA* knockdown. Among those, we identified that the protein level of MCL1 increased significantly (Fig. 5E and Supplementary Fig. 11A). Given that levels of MCL1 demonstrated a trend to anti-correlation with sensitivity to BCL2 inhibitors [30], we treated THP-1 cells with venetoclax and compared the apoptosis rates. The results indicated that *CEBPA* knockdown conferred THP-1 cells moderate resistance to venetoclax (Fig. 5F). Based on the different regulatory effects of C/EBPα-p42 and C/EBPα-p30 on DDIT3 expression, we separately examined their regulatory effects on BCL2 family proteins and cell sensitivity to venetoclax. The results indicated that C/EBPα-p30 was able to downregulate BCL2 in NB4 cells and THP-1 cells (Fig. 5G-H and Supplementary Fig. 11B-11C). Accordingly, venetoclax sensitivity was reduced by overexpression of C/EBPα-p30 and enhanced by overexpression of C/EBPα-p42 (Fig. 5I-J). Together, these data demonstrate that C/EBPα-DDIT3 mediates the altered AML cells sensitivity to BCL2 inhibition by regulating the balance of anti-apoptotic proteins.

### Activation of *DDIT3* expression synergizes with Venetoclax to enhance apoptotic death of BCL2 inhibitor-resistant NB4 cells through MCL1 inhibition in vitro and in vivo

**Fig. 6:**
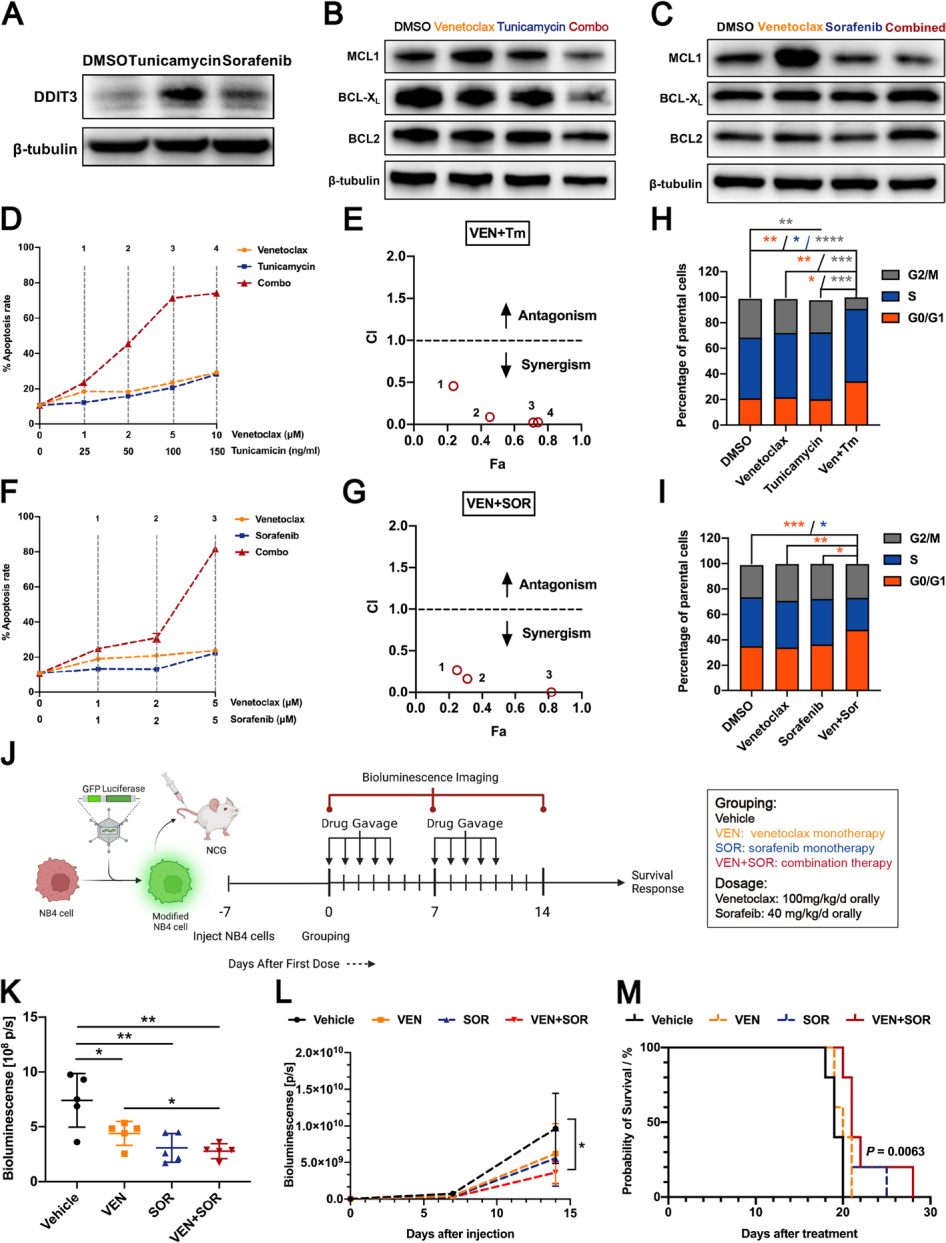
Activation of *DDIT3* expression synergizes with Venetoclax to enhance apoptotic death of BCL2 inhibitor-resistant NB4 cells through MCL1 inhibition in vitro and in vivo. **A** The protein levels of DDIT3 were increased in NB4 cells after the treatment of tunicamycin (100 ng/ml, 24 h) and sorafenib (3 μM, 24 h). **B**-**C** The protein level of MCL1 could be up-regulated by venetoclax, while down-regulated by tunicamycin (**B**) and sorafenib (**C**). The MCL1 levels of NB4 cells were further decreased by treatment with tunicamycin + venetocalx and sorafenib + venetoclax. **D** Combined treatment with tunicamycin and venetoclax resulted in increased apoptosis/cell death of NB4 cells compared to each drug alone. **E** The combination indexes (CIs) were calculated for each concentration combination of tunicamycin and venetoclax, and the CIs were all less than 1, indicating the synergistic effect. **F** Combined treatment with sorafenib and venetoclax resulted in increased apoptosis/cell death of NB4 cells compared to each drug alone. **G** The CIs were also calculated for each concentration combination of sorafenib and venetoclax, and the values were all less than 1. **H** NB4 cells treated with combination of tunicamycin (100 ng/ml, 24 h) and venetoclax (5 μM, 48 h) had a significantly increased proportion of cells in the G0/G1 phase and a significantly decreased proportion of cells in the G2/M phase compared to cells treated with a single drug or DMSO. **I** NB4 cells treated with combination of sorafenib (3 μM, 24 h) and venetoclax (5 μM, 24 h) had a significantly increased proportion of cells in the G0/G1 phase and a significantly decreased proportion of cells in the S phase compared to cells treated with a single drug or DMSO. **J** Treatment schedule and experimental set-up. **K** Quantification of bioluminescence showed the lowest tumor load in mice after treatment with venetocalx (100 mg/kg/d) + sorafenib (40 mg/kg/d) on day 7. **L** During the process of treatment, the group with co-administration of venetoclax and sorafenib continuously showed the lowest leukemia load by quantification of bioluminescence, and was the only group that showed a significantly decreased AML burden compared to vehicle group on day 14. **M** Kaplan-Meyer survival curve revealed that co-administration of venetoclax and sorafenib substantially prolonged animal survival compared to vehicle group (statistical analysis by Log-rank test, *P* = 0.0063). Data represent Mean ± SD (*n* = 3); Each animal group included 5 mice; *P < 0.05; **P < 0.01, ***P < 0.001, ****P < 0.0001

Interestingly, we found that AML cell lines insensitive to venetoclax were sensitive to tunicamycin, and vice versa. Based on the above experimental results, we speculated whether venetoclax and ER stress inducers could exert synergistic anti-leukemic effects. In addition to tunicamycin, we have also introduced another clinically approved drug, sorafenib, which was confirmed to induce ER stress in various types of cells through a mechanism independent of the MEK1/2-ERK1/2 pathway [31–33]. Firstly, we confirmed that tunicamycin and sorafenib could both activate the expression of pro-apoptotic DDIT3 in the terminal UPR signaling axis (Fig. 6A and Supplementary Fig. 12A-12B). Then, we detected the susceptibility of AML cells to ER stress inducers treatment and found negative correlation trends between *CEBPA* expression and drug-induced apoptosis (Supplementary Fig. 12C). After venetoclax treatment, MCL1 protein levels in venetoclax-resistant NB4 cells significantly increased, indicating the occurrence of further acquired resistance. However, this alteration could be abrogated by the combination with tunicamycin or sorafenib (Fig. 6B-C and Supplementary Fig. 13A-13B). MCL1 has a short half-life and is constantly degraded by proteasomes, while MCL1 T163 phosphorylation induced by p-ERK can help stabilize MCL1 [34, 35]. In order to identify the possible reason for the decrease in MCL1 protein after combination therapy, we detected the altered phosphorylation of ERK1/2 in NB4 cells after treatment with ER stress inducers monotherapy or in combination with venetoclax. As we expected, a significant suppression of p-ERK in NB4 cells was observed when treated with tunicamycin or sorafenib in combination with venetoclax (Supplementary Fig. 14A-14B). It is noteworthy that protein levels of BCL2 and BCL-X_L_ were stable after treatment with each single agent or their combination (Fig. 6B-C and Supplementary Fig. 13A-13B). Correspondingly, significantly enhanced apoptosis and cell death were observed in cells under the combined treatment of tunicamycin and venetoclax compared with each drug alone (Fig. 6D and Supplementary Fig. 15A). The synergy of tunicamycin and venetoclax is indicated by a combination index (CI) of less than 1 (0.456, 0.087, 0.022, and 0.026, for each combined concentration, respectively) (Fig. 6E). The combination of sorafenib and venetoclax also showed increased apoptosis/cell death and synergistic effects with CIs of 0.265, 0.162, and 0.001, for each combined concentration, respectively (Fig. 6F-G and Supplementary Fig. 15B). In addition, we examined the effect of the combination therapy on the cell cycle. NB4 cells treated with a combination of tunicamycin and venetoclax had a significantly increased proportion of cells in the G0/G1 phase and a significantly decreased proportion of cells in the G2/M phase compared to cells treated with a single drug or DMSO (Fig. 6H). Similar results were obtained with the combination of sorafenib and/or venetoclax for the increased G0/G1 cells and significantly decreased S phase cells (Fig. 6I). Based on our in vitro data, low transcription levels of *CEBPA* or low p42/p30 ratio helps AML cells resist to venetoclax, giving a chance to terminal UPR induction. To address whether the combination of venetoclax and a UPR activator would be synergistic against NB4 cells in vivo, we generated a xenograft model with NCG mice by inoculating luc/GFP-labeled NB4 cells (Fig. 6J). Unfortunately, the mice in groups treated with tunicamycin gavage died shortly after treatment due to drug toxicity. It was difficult to determine the appropriate dosage of tunicamycin that exerted an anti-leukemic effect while ensuring safety. Alternatively, based on previous reports, we chose sorafenib as the DDIT3 and UPR activator. As presented in Fig. 6K and Supplementary Fig. 16A, compared with treatment with vehicle, both sorafenib and venetoclax alone slowed the progression of AML on day 7 (taken as the time point of near-maximal response to the first treatment cycle), while the combination therapy group had the lowest AML burden. After the second treatment cycle (day 14), only the combination therapy group showed significantly slowed AML progression compared to the vehicle group (Fig. 6L and Supplementary Fig. 16B). Treatment was withdrawn from the day 15 onwards. According to the survival analysis, treatment with venetoclax and sorafenib as single agents modestly improved the survival of mice, whereas co-administration of venetoclax and sorafenib significantly prolonged animal survival (Fig. 6M). The mice with the longest survival time in the vehicle, venetoclax, sorafenib, and combination groups died on days 20, 21, 25, and 28, respectively (Fig. 6M). In summary, these experiments demonstrated that ER stress inducers, including tunicamycin and sorafenib, can help AML cells overcome the resistance to venetoclax by inhibition of MCL1 and cell cycle regulation.

## Discussion

The C/EBP family of TFs consists of six members—C/EBPα, C/EBPβ, C/EBPγ, C/EBPδ, C/EBPε and DDIT3 [36]. Among those, C/EBPα is the founding member initially characterized in adipogenesis and later found to be one of the most essential TFs involving myeloid differentiation [6, 37]. Interestingly, the mutual regulatory effects among members are quite common [20–22]. Here, we provided further data on the correlation between DDIT3 and C/EBPα, which are involved in the terminal UPR signaling and leukemogenesis respectively, and their pivotal role in leukemia prevalence and drug sensitivity.

UPR signaling plays an important role in maintaining the integrity of hematopoietic stem progenitor cells (HSPCs). IRE1α-XBP1, an adaptive signaling of the UPR, was shown to protect HSCs from ER stress-induced apoptosis [17]. IRE1α knockout leads to reduced reconstitution of HSCs whereas the activation of the IRE1α-XBP1 pathway confers HSCs resistance to proteotoxic stress and promotes self-renewal [17]. In addition to IRE1α-XBP1 axis, PERK-ATF4-DDIT3 axis is another UPR pathway. Under mild ER stress conditions such as amino acid deprivation, the ATF4 mediated response has a cellular protective effect and promotes the persistence of HSCs [38]. However, under severe ER stress conditions, sustained activation of the PERK-ATF4 axis leads to the expression of pro-apoptotic factor DDIT3 and confers cell sensitivity to ER stress-induced apoptosis [39]. Therefore, the regulation of stress-related genes (such as DDIT3) can protect HSPCs and progenitor cells from protein toxicity stress and improve their reconstruction ability [40]. In the present study, we revealed that in normal myeloid progenitor cells, C/EBPα-p42 directly suppresses the *DDIT3* promoter. When C/EBPα-p42 is lost in myeloid progenitor cells, cell differentiation is blocked and the DDIT3 transcript is unsealed to induce terminal UPR related cell death. Through the above mechanism, *CEBPA* maintains a steady state between survival, differentiation, and apoptosis by controlling the DDIT3 axis of the UPR, so as to survey the leukemic transformation of cells with blocked granulocytic differentiation. Therefore, the identification of C/EBPα-DDIT3 axis as a regulator of UPR signaling during myeloid differentiation helps us better understand the physiological mechanisms by which HSPCs with *CEBPA* deficiency avoid leukemia transformation.

Leukemic and preleukemic cells always experience harsh environmental conditions, including hypoxia and nutritional deprivation, owing to their rapid proliferation [41]. Therefore, it is also vital to study the regulation of UPR signaling in AML cells. Several studies have demonstrated that adaptive UPR activation mediated by common oncogenic alterations in leukemia can increase the ability of cells to cope with ER stress and restore ER homeostasis, including BCR-ABL [42], PML-RARα [43], and enhanced MYC, N-Ras, and c-Jun activity [41, 44, 45]. Among these, c-Jun directly binds to the promoters of key pro-survival UPR effectors XBP1 and ATF4 to activate their transcription and allow AML cells to overcome the damage from ER stress [44]. N-RasG12D activates IRE1α-XBP1 through the MEK-GSK3β pathway to promote survival of HSCs under ER stress [17]. Given the dual direction of the UPR to either promote cell survival or induce death, inhibiting its pro-survival arm and exaggerating its pro-apoptotic arm can be employed to target leukemia cells. ER stress induced by tunicamycin strongly increases ATO toxicity in acute promyelocytic leukemia cells, even in retinoic acid (RA)-resistant cells [46]. The triple combination of RA, tunicamycin, and ATO decreases the colony-forming capacity of primary leukemic blasts bearing the FLT3-ITD mutation, leading to AML cell death without affecting healthy HSPCs [18]. Here, we found that C/EBPα-p42 and C/EBPα-p30 regulate DDIT3 in the opposite way. A low C/EBPα p42/p30 ratio resulted in direct activation of *DDIT3* promoter. The increased expression of pro-apoptotic DDIT3 enhanced the elimination of AML cells by terminal UPR under stress conditions. This suggests that targeting the vulnerability of this kind of AML cells, such as *CEBPA*^bi^ whose C/EBPα-p42 is ineffective and C/EBPα-p30 is elevated, in the face of ER stress inducers can be a potential therapeutic strategy.

DDIT3 actively promotes cell apoptosis by suppressing anti-apoptosis gene *BCL2* and increasing the expression of pro-apoptotic members in the BCL2 family [16, 47]. As a key pathway for regulating cell survival, various small molecule inhibitors targeting the BCL2 family proteins have been developed. Among them, BCL2 inhibitor venetoclax, combined with hypomethylating agents, was initially approved by the U.S. Food and Drug Administration (FDA) in November 2018, for the treatment of patients with AML who are older or unfit for intensive chemotherapy [48]. Despite significantly improved response rates over azacitidine alone, many patients are primary refractory to venetoclax-based therapy, or relapsed subsequently due to acquired resistance [48–50]. The resistance to BCL2 inhibitors is associated with various factors, including the dependence of AML cells on BCL2 [51], cytogenetic abnormalities [49, 52–56], and changes in metabolic pathways [57, 58]. Cytogenetic abnormalities are often associated with downregulation of BCL2 and elevation of other anti-apoptotic proteins such as MCL1 and BCL-X_L_. For example, TP53 mutations in AML are associated with primary and secondary resistance of venetoclax [49, 53]. Through in-depth exploration, it has been shown that the inactivation or loss of p53 protein can lead to reduced BAX/BAK activation, ineffective MOMP during apoptosis, altered BCL2 expression and upregulated MCL1 expression, leading to resistance to venetoclax based therapies [59]. In addition, mutations in *FLT3* [54–56], *PTPN11* [52] and *KRAS* [52] can also cause venetoclax resistance by upregulating MCL1 and/or BCL-X_L_. Based on that, combination therapy is a promising strategy for overcoming venetoclax resistance and has achieved significant therapeutic effects in preclinical studies [60–63]. According to our findings, reduction of C/EBPα p42/p30 ratio in AML cells suppresses BCL2 and induces venetoclax resistance by up-regulating DDIT3. This reminds us that patients with *CEBPA*^bi^ may not benefit from venetoclax monotherapy. Due to the high sensitivity of these cells to ER stress-induced apoptosis, we hypothesized that targeting terminal UPR could be used to overcome resistance to venetoclax and achieve more significant antileukemic effect. According to our results, ER stress inducers tunicamycin and sorafenib both synergized with venetoclax to enhance apoptotic death of venetoclax-resistant NB4 cells through DDIT3 activation, MCL1 inhibition and cell cycle regulation. It is reasonable to surmise that the future of venetoclax-based therapy in AML lies in the rational combination of different drugs, and the combination of venetoclax and ER stress inducers may become a promising newcomer.

## Conclusions

**Fig. 7:**
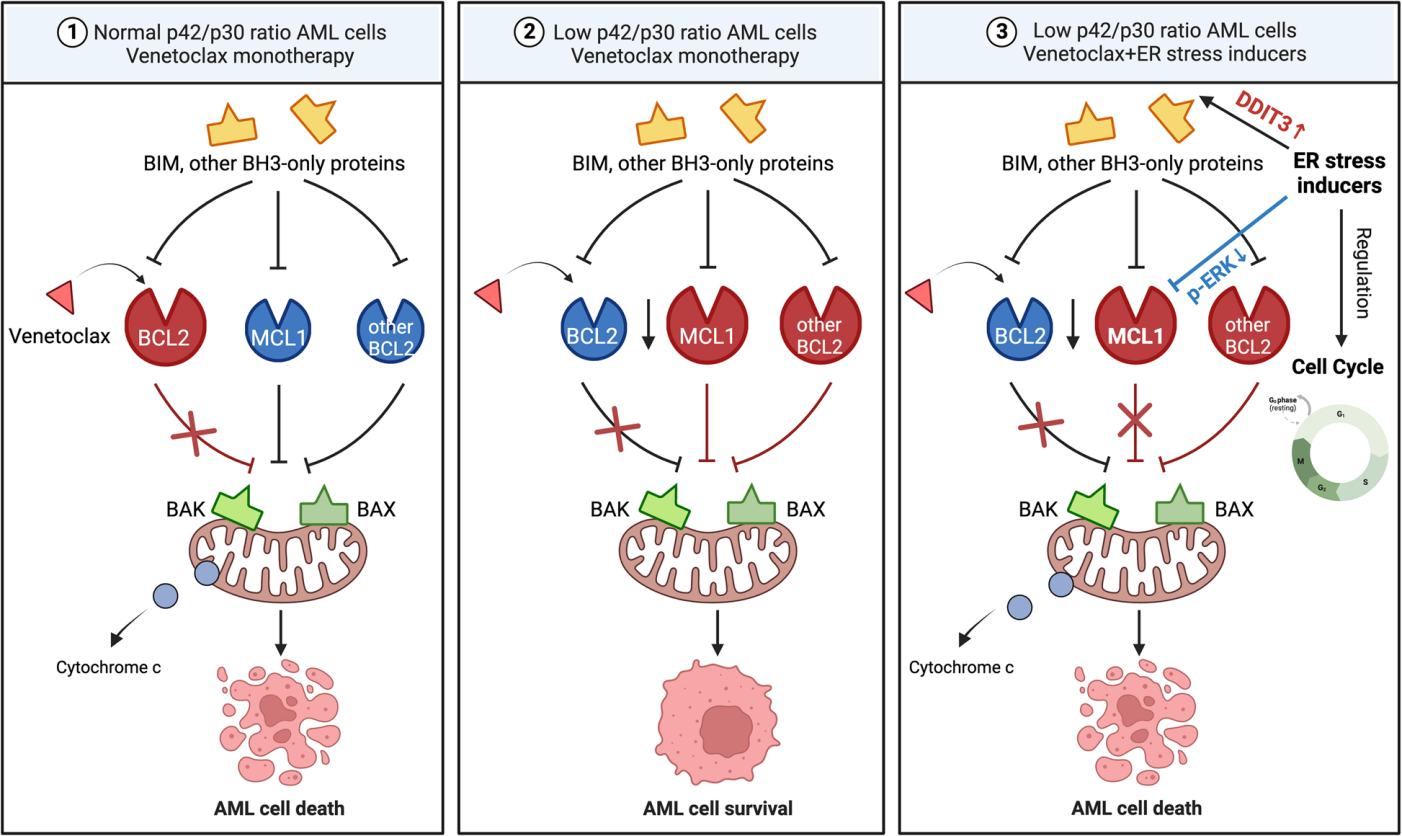
Illustration of the mechanism in a schematic format. When abnormalities in the *CEBPA* gene (e.g., *CEBPA*^bi^) in AML cells cause conversion of C/EBPα-p42 to C/EBPα-p30 isoforms, excess C/EBPα-p30 reduces the dependence of AML cells on BCL2 and increases their dependence on other anti-apoptotic proteins, thereby making them resistant to venetoclax; The combination of ER stress inducers can overcome the resistance of AML cells with low C/EBPα p42/p30 ratio to venetoclax through MCL1 inhibition and cell cycle regulation

Collectively, our results identified a critical regulation of C/EBPα on the transcription of *DDIT3* in AML cells as well as normal myeloid progenitor cells and confirmed the importance of this regulation in surveillance and elimination of AML cells. Moreover, we demonstrated that patients with AML having a low C/EBPα p42/p30 ratio (e.g., *CEBPA*^bi^) may not benefit from monotherapy with BCL2 inhibitors. This can be resolved by a combination of ER stress inducers, including tunicamycin and sorafenib (Fig. 7).

### Supplementary Information

**Additional file 1:** Supplemental Materials and Methods. **Supplementary Table 1.** Patient characteristics. **Supplementary Table 2.** Human cDNA Primers for quantitative PCR. **Supplementary Table 3.** Mouse cDNA Primers for quantitative PCR. **Supplementary Table 4.** ChIP-qPCR primer sequences for putative C/EBPα binding sites in the promoter region of *DDIT3* gene. **Supplementary Fig. 1** (A) The RNA-seq data from Beat AML project of AML patients was analyzed using cBioPortal database, and a significant negative linear correlation was observed between *CEBPA* and *DDIT3 *expression in these cases (Pearson: -0.13, *P *= 5.136e-3). (B) The microarray data from GEO (GSE38987) (https://www.ncbi.nlm.nih.gov/gds) of AML patients was analyzed, and a negative linear correlation trend was observed in these cases. **Supplementary Fig. 2** (A) Flow cytometric analysis of the apoptosis rates of AML cell lines (Kasumi-1, K562, HEL, NB4, KG-1, MOLM-13, MV4-11, THP-1, HL-60) after the treatment of tunicamycin (100 ng/ml, 48 hours). (B) Apoptosis rates of cells treated with tunicamycin (100 ng/ml) for 48 hours. The results of Kasumi-1, K562, and HEL were shown for better survival rate after treatment, which might be due to the activation of the adaptive UPR. (C) The sensitivity of AML cells to tunicamycin cytotoxicity was positively relevant to the expression level of *DDIT3* in AML cell lines. **Supplementary Fig. 3** Flow cytometric analysis revealed that compared with the negative control group, THP-1 cells with *CEBPA* knockdown displayed a significantly increased apoptosis rate after the treatment with tunicamycin (100 ng/ml, 48 hours). **Supplementary Fig. 4.** Flow cytometric analysis revealed that compared with the negative control group, 32Dcl3-shCebpa cells displayed a significantly increased apoptosis rate during granulocytic differentiation induced by G-CSF (100 ng/ml, 48 hours). **Supplementary Fig. 5.** The C/EBPα p42/p30 ratio was calculated based on the relative optical density. The Pearson's correlation test was applied to determine the correlation of the C/EBPα p42/p30 ratio and DDIT3. A negative linear correlation trend was observed between the C/EBPα p42/p30 ratio and DDIT3 at the protein level. **Supplementary Fig. 6.** (A) Flow cytometric analysis revealed that compared with the negative control group, NB4 cells with the induction of C/EBPα-p42 displayed a significantly reduced apoptosis rate after the treatment with tunicamycin (100 ng/ml, 48 hours). (B) Flow cytometric analysis revealed that compared with the negative control group, THP-1 cells with the induction of C/EBPα-p30 displayed a significantly increased apoptosis rate after the treatment with tunicamycin (100 ng/ml, 48 hours). **Supplementary Fig. 7.** (A) Other four fragments in the promoter region of *DDIT3* were deleted (schematic diagram). (B) Deletions of other four fragments in the promoter region of *DDIT3* including Chr12:57521027-57521326, Chr12:57521327-57521626, Chr12:57521627-57522036 and Chr12:57522037-57522236 did not affect the function of both C/EBPα-p42 and C/EBPα-p30. (C) Schematic diagram of C/EBPα regulating DDIT3 and outcome of UPR. In *CEBPA* wild type AML cells, C/EBPα-p42 is the dominant isoform and competitively inhibits the function of C/EBPα-p30 to keep a low basal DDIT3 level. When faced with ER stress, cells tend to initiate adaptive UPR, leading to cell survival. As for low p42/p30 ratio AML cells, overexpressed C/EBPα-p30 isoform strongly increases the basal DDIT3 level. When faced with ER stress, cells tend to initiate terminal UPR, leading to cell apoptosis. **Supplementary Fig. 8.** (A) The RNA-seq data from Beat AML project of AML patients was analyzed using cBioPortal database. A positive linear correlation was observed between *CEBPA* and *BCL2* expression in these cases, and negative linear correlations were observed between *CEBPA* and *BCL2L1* and *MCL1*. (B) The microarray data from GEO (GSE38987) (https://www.ncbi.nlm.nih.gov/gds) of AML patients was analyzed. A positive linear correlation was observed between *CEBPA* and *BCL2* expression in these cases, and negative linear correlations were observed between *CEBPA* and *BCL2L1* and *MCL1*. (C) The Pearson's correlation test was applied to determine the correlations between the protein levels of C/EBPα and BCL2, MCL1, and BCL-X_L_ in AML cell lines. A positive linear correlation was observed between C/EBPα and BCL2 expression, and negative linear correlations were observed between C/EBPα and BCL-X_L_ and MCL1. (D) The Pearson's correlation test was applied to determine the correlation between the C/EBPα p42/p30 ratio and BCL2, MCL1, and BCL-X_L_ in AML cell lines. **Supplementary Fig. 9.** Flow cytometric analysis of the apoptosis rates of AML cell lines after the treatment of venetoclax (5 μM, 48 hours) and sorafenib (3 μM, 48 hours). **Supplementary Fig. 10.** (A) The histogram of the apoptosis rates of AML cell lines after the treatment of venetoclax (5 μM, 48 hours) and sorafenib (3 μM, 48 hours). The results of Kasumi-1 were not shown because of a better survival rate was detected after venetoclax treatment. (B)-(C) The sensitivity to venetoclax cytotoxicity was positively relevant to the mRNA (B) and protein (C) levels of BCL2 in AML cell lines. **Supplementary Fig. 11.** (A) Quantitative analysis of protein levels of BCL2, BCL-X_L_ and MCL1 in THP-1 cells with *CEBPA* knockdown. (B) Quantitative analysis of protein levels of BCL2, BCL-XL and MCL1 in NB4 cells with induction of C/EBPα-p42 and C/EBPα-p30. (C) Quantitative analysis of protein levels of BCL2, BCL-X_L_ and MCL1 in THP-1 cells with induction of C/EBPα-p30. **Supplementary Fig. 12.** (A) The mRNA levels of DDIT3 were increased in NB4 cells after the treatment of tunicamycin (100 ng/ml, 24 hours) and sorafenib (3 μM, 24 hours). (B) Quantitative analysis of protein levels of DDIT3 in NB4 cells after the treatment of tunicamycin (100 ng/ml, 24 hours) and sorafenib (3 μM, 24 hours). (C) Correlation analysis using the Pearson model revealed that the mRNA levels of *CEBPA* in AML cell lines tended to be negatively correlated with tunicamycin (100 ng/ml, 48 hours) and sorafenib (3 μM, 48 hours) induced apoptosis rates. **Supplementary Fig. 13.** (A) Quantitative analysis of protein levels of BCL2, BCL-X_L_ and MCL1 in NB4 cells with combined treatment with tunicamycin and venetoclax and each drug alone. (B) Quantitative analysis of protein levels of BCL2, BCL-X_L_ and MCL1 in NB4 cells with combined treatment with sorafenib and venetoclax and each drug alone. **Supplementary Fig. 14.** (A) The protein levels of p-ERK in NB4 cells were significantly downregulated by treatment with venetoclax+tunicamycin and venetoclax+sorafenib. (B) Quantitative analysis of protein levels of p-ERK and ERK after the treatment of DMSO, venetoclax, tunicamycin, venetoclax+tunicamycin, sorafenib and venetoclax+sorafenib. **Supplementary Fig. 15.** (A)-(B) Flow cytometric analysis revealed that combined treatment with tunicamycin (100 ng/ml) and venetoclax (5 μM) resulted in increased apoptosis/cell death of NB4 cells compared to either drug alone (A), and combined treatment with sorafenib (5 μM) and venetoclax (5 μM) also resulted in increased apoptosis/cell death compared to either drug alone (B). **Supplementary Fig. 16.** (A) BLI of leukemia growth on day 7 and the histogram. (B) BLI of leukemia growth on day 14 and the histogram.

## Data Availability

The datasets supporting the conclusions of this article are included within the article and its additional files.

## Abbreviations

AML: Acute myeloid leukemia
ATF4: Activating transcription factor 4
ATO: Arsenic trioxide
C/EBPα: CCAAT-enhancer-binding protein α
*CEBPA*^bi^: *CEBPA* Biallelically mutated
CI: Combination index
DDIT3: DNA damage-inducible transcript 3
ER: Endoplasmic reticulum
HSCs/HSPCs: Hematopoietic stem/ progenitor cells (HSPCs)
IRE1α: Inositol-requiring enzyme 1α
NGS: Next-generation sequencing
ORF: Open reading frame
PERK: Pancreatic endoplasmic reticulum kinase
Pre-LSCs: Preleukemic stem cells
RA: Retinoic acid
TADs: Transactivation domains
TFs: Transcription factors
UPR: Unfolded protein response
XBP1s: The spliced form of X-box binding protein 1

## References

[1] Newell LF, Cook RJ. Advances in acute myeloid leukemia. BMJ. 2021;375:n2026. 10.1136/bmj.n2026 34615640

[2] Döhner H, Weisdorf DJ, Bloomfield CD (2015). Acute myeloid leukemia. Longo DL, editor. N Engl J Med..

[3] Bullinger L, Döhner K, Döhner H (2017). Genomics of acute myeloid leukemia diagnosis and pathways. JCO.

[4] DiNardo CD, Perl AE (2019). Advances in patient care through increasingly individualized therapy. Nat Rev Clin Oncol.

[5] Arber DA, Orazi A, Hasserjian R, Thiele J, Borowitz MJ, Le Beau MM (2016). The 2016 revision to the World Health Organization classification of myeloid neoplasms and acute leukemia. Blood.

[6] Avellino R, Delwel R (2017). Expression and regulation of C/EBPα in normal myelopoiesis and in malignant transformation. Blood.

[7] Nie Y, Su L, Li W, Gao S (2021). Novel insights of acute myeloid leukemia with CEBPA deregulation: heterogeneity dissection and re-stratification. Crit Rev Oncol Hematol.

[8] Calkhoven CF, Müller C, Leutz A (2000). Translational control of C/EBPalpha and C/EBPbeta isoform expression. Genes Dev.

[9] Lin FT, MacDougald OA, Diehl AM, Lane MD (1993). A 30-kDa alternative translation product of the CCAAT/ENHANCER binding protein alpha message: transcriptional activator lacking antimitotic activity. Proc Natl Acad Sci U S A.

[10] Lincoln AJ, Monczak Y, Williams SC, Johnson PF (1998). Inhibition of CCAAT/ENHANCER-binding protein alpha and beta translation by upstream open reading frames. J Biol Chem.

[11] El-Sharkawi D, Sproul D, Allen CG, Feber A, Wright M, Hills RK (2018). Variable outcome and methylation status according to *CEBPA* mutant type in double-mutated acute myeloid leukemia patients and the possible implications for treatment. Haematologica.

[12] Wouters BJ, Löwenberg B, Erpelinck-Verschueren CAJ, van Putten WLJ, Valk PJM, Delwel R (2009). Double CEBPA mutations, but not single CEBPA mutations, define a subgroup of acute myeloid leukemia with a distinctive gene expression profile that is uniquely associated with a favorable outcome. Blood.

[13] Döhner H, Wei AH, Appelbaum FR, Craddock C, DiNardo CD, Dombret H (2022). Diagnosis and management of AML in adults: 2022 recommendations from an international expert panel on behalf of the ELN. Blood.

[14] Taube F, Georgi JA, Kramer M, Stasik S, Middeke JM, Röllig C (2022). CEBPA mutations in 4708 patients with acute myeloid leukemia: differential impact of bZIP and TAD mutations on outcome. Blood.

[15] Féral K, Jaud M, Philippe C, Di Bella D, Pyronnet S, Rouault-Pierre K (2021). ER stress and unfolded protein response in leukemia: friend, foe, or both?. Biomolecules.

[16] Oakes SA, Papa FR (2015). The role of endoplasmic reticulum stress in human pathology. Annu Rev Pathol.

[17] Liu L, Zhao M, Jin X, Ney G, Yang KB, Peng F (2019). Adaptive endoplasmic reticulum stress signalling via IRE1α-XBP1 preserves self-renewal of haematopoietic and pre-leukaemic stem cells. Nat Cell Biol.

[18] Masciarelli S, Capuano E, Ottone T, Divona M, Lavorgna S, Liccardo F (2019). Retinoic acid synergizes with the unfolded protein response and oxidative stress to induce cell death in FLT3-ITD+ AML. Blood Adv.

[19] Oyadomari S, Mori M (2004). Roles of CHOP/GADD153 in endoplasmic reticulum stress. Cell Death Differ.

[20] Parkin SE, Baer M, Copeland TD, Schwartz RC, Johnson PF (2002). Regulation of CCAAT/ENHANCER-binding protein (C/EBP) activator proteins by heterodimerization with C/EBPgamma (IG/EBP). J Biol Chem.

[21] Cooper C, Henderson A, Artandi S, Avitahl N, Calame K (1995). IG/EBP (C/EBP gamma) is a transdominant negative inhibitor of C/EBP family transcriptional activators. Nucleic Acids Res.

[22] Alberich-Jordà M, Wouters B, Balastik M, Shapiro-Koss C, Zhang H, Di Ruscio A (2012). C/EBPγ deregulation results in differentiation arrest in acute myeloid leukemia. J Clin Invest.

[23] Liu M, Du M, Yu J, Qian Z, Gao Y, Pan W (2022). CEBPA mutants down-regulate AML cell susceptibility to NK-mediated lysis by disruption of the expression of NKG2D ligands, which can be restored by LSD1 inhibition. Oncoimmunology.

[24] Livak KJ, Schmittgen TD (2001). Analysis of relative gene expression data using real-time quantitative PCR and the 2(-Delta Delta C(T)) method. Methods.

[25] Wu J, Chen S, Liu H, Zhang Z, Ni Z, Chen J (2018). Tunicamycin specifically aggravates ER stress and overcomes chemoresistance in multidrug-resistant gastric cancer cells by inhibiting N-glycosylation. J Exp Clin Cancer Res.

[26] Pulikkan JA, Tenen DG, Behre G (2017). C/EBPα deregulation as a paradigm for leukemogenesis. Leukemia.

[27] Heyes E, Schmidt L, Manhart G, Eder T, Proietti L, Grebien F (2021). Identification of gene targets of mutant C/EBPα reveals a critical role for MSI2 in CEBPA-mutated AML. Leukemia.

[28] Hughes JM, Legnini I, Salvatori B, Masciarelli S, Marchioni M, Fazi F (2015). C/EBPα-p30 protein induces expression of the oncogenic long non-coding RNA UCA1 in acute myeloid leukemia. Oncotarget.

[29] Jakobsen JS, Laursen LG, Schuster MB, Pundhir S, Schoof E, Ge Y (2019). Mutant CEBPA directly drives the expression of the targetable tumor-promoting factor CD73 in AML. Sci Adv..

[30] Sullivan GP, Flanagan L, Rodrigues DA, Ní Chonghaile T (2022). The path to venetoclax resistance is paved with mutations, metabolism, and more. Sci Transl Med..

[31] Holz MS, Janning A, Renné C, Gattenlöhner S, Spieker T, Bräuninger A (2013). Induction of endoplasmic reticulum stress by sorafenib and activation of NF-κB by lestaurtinib as a novel resistance mechanism in Hodgkin lymphoma cell lines. Mol Cancer Ther.

[32] Shi Y-H, Ding Z-B, Zhou J, Hui B, Shi G-M, Ke A-W (2011). Targeting autophagy enhances sorafenib lethality for hepatocellular carcinoma via ER stress-related apoptosis. Autophagy.

[33] Wang X, Hu R, Song Z, Zhao H, Pan Z, Feng Y (2022). Sorafenib combined with STAT3 knockdown triggers ER stress-induced HCC apoptosis and cGAS-STING-mediated anti-tumor immunity. Cancer Lett.

[34] Senichkin VV, Streletskaia AY, Gorbunova AS, Zhivotovsky B, Kopeina GS (2020). Saga of Mcl-1: regulation from transcription to degradation. Cell Death Differ.

[35] Wu X, Luo Q, Liu Z (2020). Ubiquitination and deubiquitination of MCL1 in cancer: deciphering chemoresistance mechanisms and providing potential therapeutic options. Cell Death Dis.

[36] Tolomeo M, Grimaudo S (2020). The, “Janus” role of C/EBPs family members in cancer progression. Int J Mol Sci.

[37] Ohlsson E, Schuster MB, Hasemann M, Porse BT (2016). The multifaceted functions of C/EBPα in normal and malignant haematopoiesis. Leukemia.

[38] van Galen P, Mbong N, Kreso A, Schoof EM, Wagenblast E, Ng SWK (2018). Integrated stress response activity Marks stem cells in Normal hematopoiesis and leukemia. Cell Rep.

[39] Marciniak SJ, Yun CY, Oyadomari S, Novoa I, Zhang Y, Jungreis R (2004). CHOP induces death by promoting protein synthesis and oxidation in the stressed endoplasmic reticulum. Genes Dev.

[40] van Galen P, Kreso A, Mbong N, Kent DG, Fitzmaurice T, Chambers JE (2014). The unfolded protein response governs integrity of the haematopoietic stem-cell pool during stress. Nature.

[41] Khateb A, Ronai ZA (2020). Unfolded protein response in leukemia: from basic understanding to therapeutic opportunities. Trends Cancer.

[42] Piwocka K, Vejda S, Cotter TG, O’Sullivan GC, McKenna SL (2006). Bcr-Abl reduces endoplasmic reticulum releasable calcium levels by a Bcl-2-independent mechanism and inhibits calcium-dependent apoptotic signaling. Blood.

[43] Khan MM, Nomura T, Chiba T, Tanaka K, Yoshida H, Mori K (2004). The fusion oncoprotein PML-RARalpha induces endoplasmic reticulum (ER)-associated degradation of N-CoR and ER stress. J Biol Chem.

[44] Zhou C, Martinez E, Di Marcantonio D, Solanki-Patel N, Aghayev T, Peri S (2017). JUN is a key transcriptional regulator of the unfolded protein response in acute myeloid leukemia. Leukemia.

[45] Huiting LN, Samaha Y, Zhang GL, Roderick JE, Li B, Anderson NM (2018). UFD1 contributes to MYC-mediated leukemia aggressiveness through suppression of the proapoptotic unfolded protein response. Leukemia.

[46] Masciarelli S, Capuano E, Ottone T, Divona M, De Panfilis S, Banella C (2018). Retinoic acid and arsenic trioxide sensitize acute promyelocytic leukemia cells to ER stress. Leukemia.

[47] Hetz C, Zhang K, Kaufman RJ (2020). Mechanisms, regulation and functions of the unfolded protein response. Nat Rev Mol Cell Biol.

[48] Dhakal P, Bates M, Tomasson MH, Sutamtewagul G, Dupuy A, Bhatt VR. Acute myeloid leukemia resistant to venetoclax-based therapy: what does the future hold? Blood Rev. 2023;59:101036. 10.1016/j.blre.2022.101036 36549969

[49] DiNardo CD, Jonas BA, Pullarkat V, Thirman MJ, Garcia JS, Wei AH (2020). Azacitidine and venetoclax in previously untreated acute myeloid leukemia. N Engl J Med.

[50] Wei AH, Montesinos P, Ivanov V, DiNardo CD, Novak J, Laribi K (2020). Venetoclax plus LDAC for newly diagnosed AML ineligible for intensive chemotherapy: a phase 3 randomized placebo-controlled trial. Blood.

[51] Pei S, Pollyea DA, Gustafson A, Stevens BM, Minhajuddin M, Fu R (2020). Monocytic subclones confer resistance to venetoclax-based therapy in patients with acute myeloid leukemia. Cancer Discov.

[52] Zhang H, Nakauchi Y, Köhnke T, Stafford M, Bottomly D, Thomas R (2020). Integrated analysis of patient samples identifies biomarkers for venetoclax efficacy and combination strategies in acute myeloid leukemia. Nat Cancer.

[53] DiNardo CD, Tiong IS, Quaglieri A, MacRaild S, Loghavi S, Brown FC (2020). Molecular patterns of response and treatment failure after frontline venetoclax combinations in older patients with AML. Blood.

[54] Rahmani M, Aust MM, Attkisson E, Williams DC, Ferreira-Gonzalez A, Grant S (2012). Inhibition of Bcl-2 antiapoptotic members by obatoclax potently enhances sorafenib-induced apoptosis in human myeloid leukemia cells through a Bim-dependent process. Blood.

[55] Ma J, Zhao S, Qiao X, Knight T, Edwards H, Polin L (2019). Inhibition of Bcl-2 synergistically enhances the antileukemic activity of midostaurin and gilteritinib in preclinical models of FLT3-mutated acute myeloid leukemia. Clin Cancer Res.

[56] Singh Mali R, Zhang Q, DeFilippis RA, Cavazos A, Kuruvilla VM, Raman J (2021). Venetoclax combines synergistically with FLT3 inhibition to effectively target leukemic cells in FLT3-ITD+ acute myeloid leukemia models. Haematologica.

[57] Jones CL, Stevens BM, Pollyea DA, Culp-Hill R, Reisz JA, Nemkov T (2020). Nicotinamide metabolism mediates resistance to venetoclax in relapsed acute myeloid leukemia stem cells. Cell Stem Cell.

[58] Stevens BM, Jones CL, Pollyea DA, Culp-Hill R, D’Alessandro A, Winters A (2020). Fatty acid metabolism underlies venetoclax resistance in acute myeloid leukemia stem cells. Nat Cancer.

[59] Nechiporuk T, Kurtz SE, Nikolova O, Liu T, Jones CL, D’Alessandro A (2019). The TP53 apoptotic network is a primary mediator of resistance to BCL2 inhibition in AML cells. Cancer Discov.

[60] Lehmann C, Friess T, Birzele F, Kiialainen A, Dangl M (2016). Superior anti-tumor activity of the MDM2 antagonist idasanutlin and the Bcl-2 inhibitor venetoclax in p53 wild-type acute myeloid leukemia models. J Hematol Oncol.

[61] Ramsey HE, Fischer MA, Lee T, Gorska AE, Arrate MP, Fuller L (2018). A novel MCL1 inhibitor combined with venetoclax rescues venetoclax-resistant acute myelogenous leukemia. Cancer Discov.

[62] Carter BZ, Mak PY, Tao W, Warmoes M, Lorenzi PL, Mak D (2022). Targeting MCL-1 dysregulates cell metabolism and leukemia-stroma interactions and resensitizes acute myeloid leukemia to BCL-2 inhibition. Haematologica.

[63] Han L, Zhang Q, Dail M, Shi C, Cavazos A, Ruvolo VR (2020). Concomitant targeting of BCL2 with venetoclax and MAPK signaling with cobimetinib in acute myeloid leukemia models. Haematologica.

