# Supplementary material for: C/EBPα-p30 confers AML cell susceptibility to the terminal unfolded protein response and resistance to Venetoclax by activating DDIT3 transcription
Source: J Exp Clin Cancer Res. 2024 Mar 13;43:79. doi: 10.1186/s13046-024-02975-3 (PMC10936103; doi:10.1186/s13046-024-02975-3)
Supplement: Supplementary file 1 — Additional file 1: Supplemental Materials and Methods. Supplementary Table 1. Patient characteristics. Supplementary Table 2. Human cDNA Primers for quantitative PCR. Supplementary Table 3. Mouse cDNA Primers for quantitative PCR. Supplementary Table 4. ChIP-qPCR primer sequences for putative C/EBPα binding sites in the promoter region of DDIT3 gene. Supplementary Fig. 1 (A) The RNA-seq data from Beat AML project of AML patients was analyzed using cBioPortal database, and a significant negative linear correlation was observed between CEBPA and DDIT3 expression in these cases (Pearson: -0.13, P = 5.136e-3). (B) The microarray data from GEO (GSE38987) (https://www.ncbi.nlm.nih.gov/gds) of AML patients was analyzed, and a negative linear correlation trend was observed in these cases. Supplementary Fig. 2 (A) Flow cytometric analysis of the apoptosis rates of AML cell lines (Kasumi-1, K562, HEL, NB4, KG-1, MOLM-13, MV4-11, THP-1, HL-60) after the treatment of tunicamycin (100 ng/ml, 48 hours). (B) Apoptosis rates of cells treated with tunicamycin (100 ng/ml) for 48 hours. The results of Kasumi-1, K562, and HEL were shown for better survival rate after treatment, which might be due to the activation of the adaptive UPR. (C) The sensitivity of AML cells to tunicamycin cytotoxicity was positively relevant to the expression level of DDIT3 in AML cell lines. Supplementary Fig. 3 Flow cytometric analysis revealed that compared with the negative control group, THP-1 cells with CEBPA knockdown displayed a significantly increased apoptosis rate after the treatment with tunicamycin (100 ng/ml, 48 hours). Supplementary Fig. 4. Flow cytometric analysis revealed that compared with the negative control group, 32Dcl3-shCebpa cells displayed a significantly increased apoptosis rate during granulocytic differentiation induced by G-CSF (100 ng/ml, 48 hours). Supplementary Fig. 5. The C/EBPα p42/p30 ratio was calculated based on the relative optical density. The Pearson's correlation test [file 13046_2024_2975_MOESM1_ESM.docx]

**Supplementary Information for**

**C/EBPα-p30 confers AML cell susceptibility to the terminal unfolded protein response and resistance to Venetoclax by activating DDIT3 transcription**

Mengbao Du^#^, Mowang Wang^#^, Meng Liu^#^, Shan Fu, Yu Lin, Yankun Huo, Jian Yu, Xiaohong Yu, Chong Wang^*^, Haowen Xiao^*^, Limengmeng Wang^*^

# These authors have contributed equally to this work and share first authorship.

*Correspondence:

**Limengmeng Wang**, Ph.D., Bone Marrow Transplantation Center, The First Affiliated Hospital, Zhejiang University School of Medicine, No. 79 Qingchun Rd., Hangzhou, 310003, Zhejiang province, P R China. Email: wanglmmzx@zju.edu.cn

**Haowen Xiao**, Ph.D., Department of Hematology, Sir Run Run Shaw Hospital, Zhejiang University School of Medicine, No. 3 East Qingchun Rd., Hangzhou, 310003, Zhejiang province, P R China. Email: [haowenxiaoxiao@zju.edu.cn](mailto:haowenxiaoxiao@zju.edu.cn)

**Chong Wang**, Ph.D., Hematology Department, The First Affiliated Hospital of Zhengzhou University, No. 1 Jianshe Dong Rd., Zhengzhou, 450000, Henan province, P R China. Email: fccwangc@zzu.edu.cn

**1. Supplemental Materials and Methods**

**2. Supplementary Table S1 to S4**

**3. Supplementary Figure S1 to S16**

1. **Supplemental Materials and Methods**

**Plasmids and lentivirus infection**

GFP-tagged ORF clone of the Homo sapiens full-length *CEBPA* coding sequence (*CEBPA*, 1077 bp, NM_004364.2) (hereafter called pCMV6-AC-GFP-C/EBPα-p42) was purchased from ORIGENE Technologies (Beijing, China). We used pCMV6-AC-GFP-C/EBPα-p42 to generate an N-terminal truncated mutation, represented by the p30 isoform (C/EBPα-p30). The two C/EBPα isoforms were further subcloned into Flag-tagged p3×FLAG-Myc-CMV (Addgene, Watertown, MA, USA) and pHIV7/SFFV-GFP lentiviral plasmids (a gift from Prof. Jiing-Kuan Yee, City of Hope National Medical Center). The *CEBPA* short hairpin RNA (shRNA) library was subcloned into the pGLV3/H1/GFP&Puro lentiviral plasmid. The *CEBPA* shRNA sequence was as follows: 5’-CAACGTGGAGACGCAACAGAA-3’.

The transfection efficiency was >80%. After transfection, *CEBPA* mRNA levels were detected by quantitative real-time PCR, and C/EBPα protein levels were detected by western immunoblotting.

**Quantitative real-time PCR**

Cell samples were lysed using TRIzol (Invitrogen, Waltham, MA, USA) and total RNA was extracted using the phenol-chloroform method. cDNA was transcribed using a reverse transcription kit (Vazyme Biotech, Nanjing, China), according to the manufacturer’s protocol. PCR was performed using a quantitative PCR kit (Vazyme Biotech) and a fluorogenic quantitative PCR instrument (Roche, Germany). Sequences of the primers used are listed in **Supplementary Table 2-3**.

**Western immunoblotting**

Protein lysates were generated by counting cells with trypan blue staining and lysed in RIPA non-denaturing lysis buffer containing phenylmethylsulfonyl fluoride (PMSF) and a proteinase inhibitor cocktail (Beyotime, Shanghai, China). Protein concentrations in the cell lysates were measured using a Pierce BCA Protein Assay Kit (Thermo Fisher Scientific, Schwerte, Germany). Samples were boiled for 10 min in sample buffer containing bromophenol blue and 1×β-Mercaptoethanol, and equal amounts of protein were loaded onto 10% polyacrylamide gels/SDS-PAGE and electrophoresis was performed (Bio-Rad, CA, USA). The proteins were then transferred to a PVDF membrane, which was blocked using 5% non-fat milk for 60 min. The membrane was then incubated with the following primary antibodies at a 1:1000 dilution: anti-C/EBPα (Abcam, USA, ab40764), anti-DDIT3 (Cell Signaling Technology, USA, #2895), anti-MCL1 (Cell Signaling Technology, #5453), anti-BCL-X_L_ (Cell Signaling Technology, #2764), anti-BCL2 (Abcam, ab182858), anti-p-ERK (T202/Y204) (Cell Signaling Technology, #4370), anti-ERK1/2 (Cell Signaling Technology, #4695) and anti-β-tubulin (TransGen Biotech, China, HC101-01). After washing three times with TBST, the membrane was incubated with goat anti-mouse or anti-rabbit-HRP secondary antibody at 1:5000 (Santa Cruz Biotechnology, TX, USA). Stripping buffer (Beyotime) was applied for the reuse of membrane with transferred protein. The blots were visualized using ECL Western Blotting Detection Reagent (Millipore, Darmstadt, Germany) and scanned using a FluorChem E system (ProteinSimple, CA, USA). The relative expression levels of proteins were statistically calculated using ImageJ with n = 3 or more independent biological replicates, normalized to β-tubulin.

**Chromatin immunoprecipitation coupled with high-throughput sequencing (ChIP-seq) and ChIP-qPCR**

As for ChIP-qPCR assay, 1×10^7^ HEK293T cells transfected with wild-type C/EBPα expression plasmids (p3×FLAG-Myc-CMV-C/EBPα-p42 and p30) and empty vector were harvested and processed with the SimpleChiP® Enzymatic Chromatin IP Kit (Agarose Beads) (Cell Signaling Technology, #9002) according to the manufacturer’s instructions. Immunoprecipitation was performed with rabbit anti-FLAG® M2 (Cell Signaling Technology, #14793), anti-Histone H3 (D2B12) XP® (Cell Signaling Technology, #4620) and IgG control antibodies (Cell Signaling Technology, # 2729). The antibody-bound chromatin was captured with Protein-G sepharose beads, washed, de-cross-linked and precipitated. Libraries were sequenced following the protocol provided by the I NEXTFLEX® ChIP-Seq Library Prep Kit for Illumina® Sequencing (NOVA-5143, Bioo Scientific, Austin, TX) and sequenced on Illumina Xten using the PE 150 method. Sequencing reads were mapped to the reference genome assembly (hg38) using Bwa (version 0.7.15), and peak calling was performed using MACS2 software (version 2.1.1.20160309).

The relative amounts of chromatin immunoprecipitated by either isotype control or FLAG mAb were quantified by real-time PCR with specific primers for C/EBPα binding sites in *DDIT3* gene promoter, respectively. Sequences of primers used for ChIP-qPCR are listed in **Supplementary Table 4**.

**Luciferase reporter assays**

For luciferase assays, HEK293T cells at 50% confluency were transfected into 24-well plates with *DDIT3* promoter luciferase reporter (psiCHECK2-DDIT3 promoter) or empty vector control (psiCHECK2), with pCMV6-AC-GFP-C/EBPα-p42 and p30 expression plasmids or pCMV6-AC-GFP empty vectors. Cells were lysed after 48 hours and luciferase activity was assessed using a Dual-Luciferase Reporter Assay System (Vazyme Biotech). Optical density (OD) values were detected using SpectraMax iD3 and iD5 Multi-Mode Microplate Readers (Molecular Devices, San Jose, USA).

We also constructed several plasmids expressing the mutated sequence of *DDIT3* promoter luciferase reporter to confirm C/EBPα motif signatures in the promoter region of *DDIT3*. The psiCHECK2 plasmid simultaneously expressed the Renilla luciferase reporter and the firefly luciferase reference. Assays were performed in at least three independent experiments.

1. **Supplementary Tables**

**Supplementary Table 1.** Patient characteristics

| **Pt No** | **Disease Status** | **Age** | **Sex** | **Diagnosis** | **FAB** | **Genetic Abnormalities** |
| --- | --- | --- | --- | --- | --- | --- |
| 1 | Newly diagnosed | 34 | Female | AML | M3 | *PML-RARA*  *FLT3-ITD* |
| 2 | Newly diagnosed | 14 | Female | AML | M2 | not detected |
| 3 | Newly diagnosed | 32 | Female | AML | M2 | not detected |
| 4 | Newly diagnosed | 49 | Female | AML | M5 | *DNMT3A*  *NPM1*  *FLT3-ITD* |
| 5 | Newly diagnosed | 42 | Male | AML | M2 | *FLT3-ITD* |
| 6 | Newly diagnosed | 14 | Male | AML | M1 | *PHF6*  *CEBPA* (N-terminal) |
| 7 | Newly diagnosed | 65 | Male | AML | M5 | *ZRSR2* |

**Supplementary Table 2.** Human cDNA Primers for quantitative PCR.

| **Gene** | **Primer pairs** | |
| --- | --- | --- |
| *CEBPA* | F | AAGAACAGCAACGAGTACC |
|  | R | GGTCATTGTCACTGGTCAG |
| *DDIT3* | F | AGAACCAGGAAACGGAAACAGA |
|  | R | TCTCCTTCATGCGCTGCTTT |
| *GRP78* | F | GAAAGAAGGTTACCCATGCAGT |
|  | R | CAGGCCATAAGCAATAGCAGC |
| *GRP94* | F | CCAGTTTGGTGTCGGTTTCTAT |
|  | R | CTGGGTATCGTTGTTGTGTTTTG |
| *ATF4* | F | CTCCGGGACAGATTGGATGTT |
|  | R | GGCTGCTTATTAGTCTCCTGGAC |
| *XBP1s* | F | CTGAGTCCGCAGCAGGTG |
|  | R | CTCTGGGGAAGGGCATTTGA |
| *XBP1u* | F | CAGACTACGTGCACCTCTGC |
|  | R | GGGTCCTTCTGGGTAGACCT |
| *BCL2* | F | CTTTGAGTTCGGTGGGGTCAT |
|  | R | GCCAGGAGAAATCAAACAGAGG |
| *BCL2L1* | F | GACTGAATCGGAGATGGAGACC |
|  | R | GCAGTTCAAACTCGTCGCCT |
| *MCL1* | F | TGCTTCGGAAACTGGACATCA |
|  | R | TAGCCACAAAGGCACCAAAAG |
| *GAPDH* | F | AGAAGGCTGGGGCTCATTTG |
|  | R | AGGGGCCATCCACAGTCTTC |

**Supplementary Table 3.** Mouse cDNA Primers for quantitative PCR.

| ***Gene*** | **Primer pairs** | |
| --- | --- | --- |
| *Cebpa* | F | GCGGGAACGCAACAACATC |
|  | R | GTCACTGGTCAACTCCAGCAC |
| *Ddit3* | F | CTGGAAGCCTGGTATGAGGAT |
|  | R | CAGGGTCAAGAGTAGTGAAGGT |
| *Grp78* | F | GCATCACGCCGTCGTATGT |
|  | R | ATTCCAAGTGCGTCCGATGAG |
| *Grp94* | F | TCGTCAGAGCTGATGATGAAGT |
|  | R | GCGTTTAACCCATCCAACTGAAT |
| *Atf4* | F | CTCTTGACCACGTTGGATGAC |
|  | R | CAACTTCACTGCCTAGCTCTAAA |
| *Xbp1s* | F | GGTCTGCTGAGTCCGCAGCAGG |
|  | R | GAAAGGGAGGCTGGTAAGGAAC |
| *Xbp1u* | F | TGAGAACCAGGAGTTAAGAACACGC |
|  | R | CACATAGTCTGAGTGCTGCGG |
| *Gapdh* | F | TGGAAAGCTGTGGCGTGAT |
|  | R | TGCTTCACCACCTTCTTGAT |
| *Mpo* | F | GCTCCGCCCGCATTCCTTGT |
|  | R | TTGAGCTGTGTGGCCAGCCG |
| *Elane* | F | CAGGCATCTGCTTCGGGGAC |
|  | R | AGGGGCGAAGGCATCTGGGT |
| *Prtn3* | F | AGCTACCCATCCCCCAAG |
|  | R | TCGTGCCCACCTACAATCTT |
| S16 | F | ATATTCGGGTCCGTGTGAAG |
|  | R | CTTGGAGGCTTCATCCACAT |

**Supplementary Table 4.** ChIP-qPCR primer sequences for putative C/EBPα binding sites in the promoter region of *DDIT3* gene.

| **Region** | **Primer pairs** | |
| --- | --- | --- |
| Peak 1 | F | CTCCGTGAAGCCTCGTGAC |
|  | R | CGTCGCTCCCTCTCGCTA |
| Peak 2 | F | CAGCTTCTGGGGGAGACAAG |
|  | R | CAACGGCTATCAGCCTTGGT |
| *Upstream site 1* | F | CGACAATCCCAGTGGATGGA |
|  | R | CCTCCCAGCTAATGGGCAC |
| *Upstream site 2* | F | AGCAGCCTAACCAAAGAC |
|  | R | CTGTTACATATCCTGTCATACC |

1. **Supplemental Figure Legends**

**Supplementary Fig. 1** (A) The RNA-seq data from Beat AML project of AML patients was analyzed using cBioPortal database, and a significant negative linear correlation was observed between *CEBPA* and *DDIT3* expression in these cases (Pearson: -0.13, *P* = 5.136e-3). (B) The microarray data from GEO (GSE38987) (https://www.ncbi.nlm.nih.gov/gds) of AML patients was analyzed, and a negative linear correlation trend was observed in these cases.

**
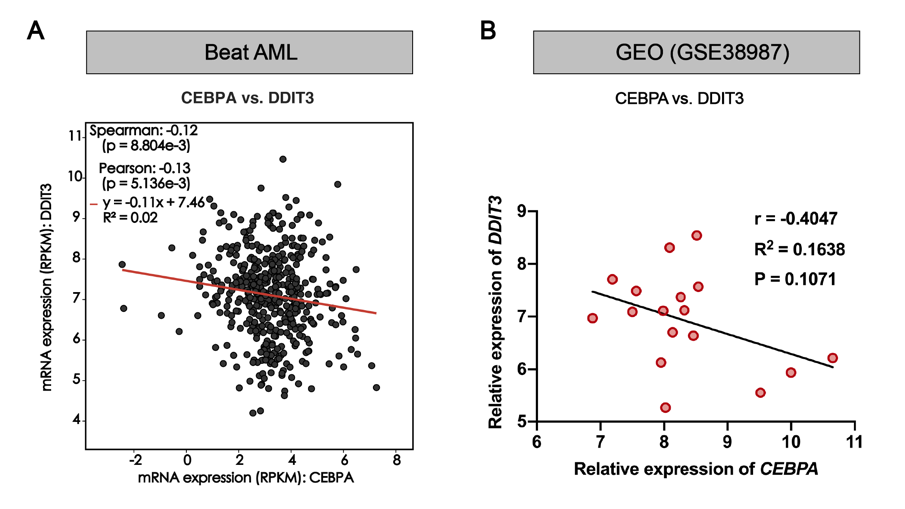
**

**Supplementary Fig. 1**

**Supplementary Fig. 2** (A) Flow cytometric analysis of the apoptosis rates of AML cell lines (Kasumi-1, K562, HEL, NB4, KG-1, MOLM-13, MV4-11, THP-1, HL-60) after the treatment of tunicamycin (100 ng/ml, 48 hours). (B) Apoptosis rates of cells treated with tunicamycin (100 ng/ml) for 48 hours. The results of Kasumi-1, K562, and HEL were shown for better survival rate after treatment, which might be due to the activation of the adaptive UPR. (C) The sensitivity of AML cells to tunicamycin cytotoxicity was positively relevant to the expression level of *DDIT3* in AML cell lines.


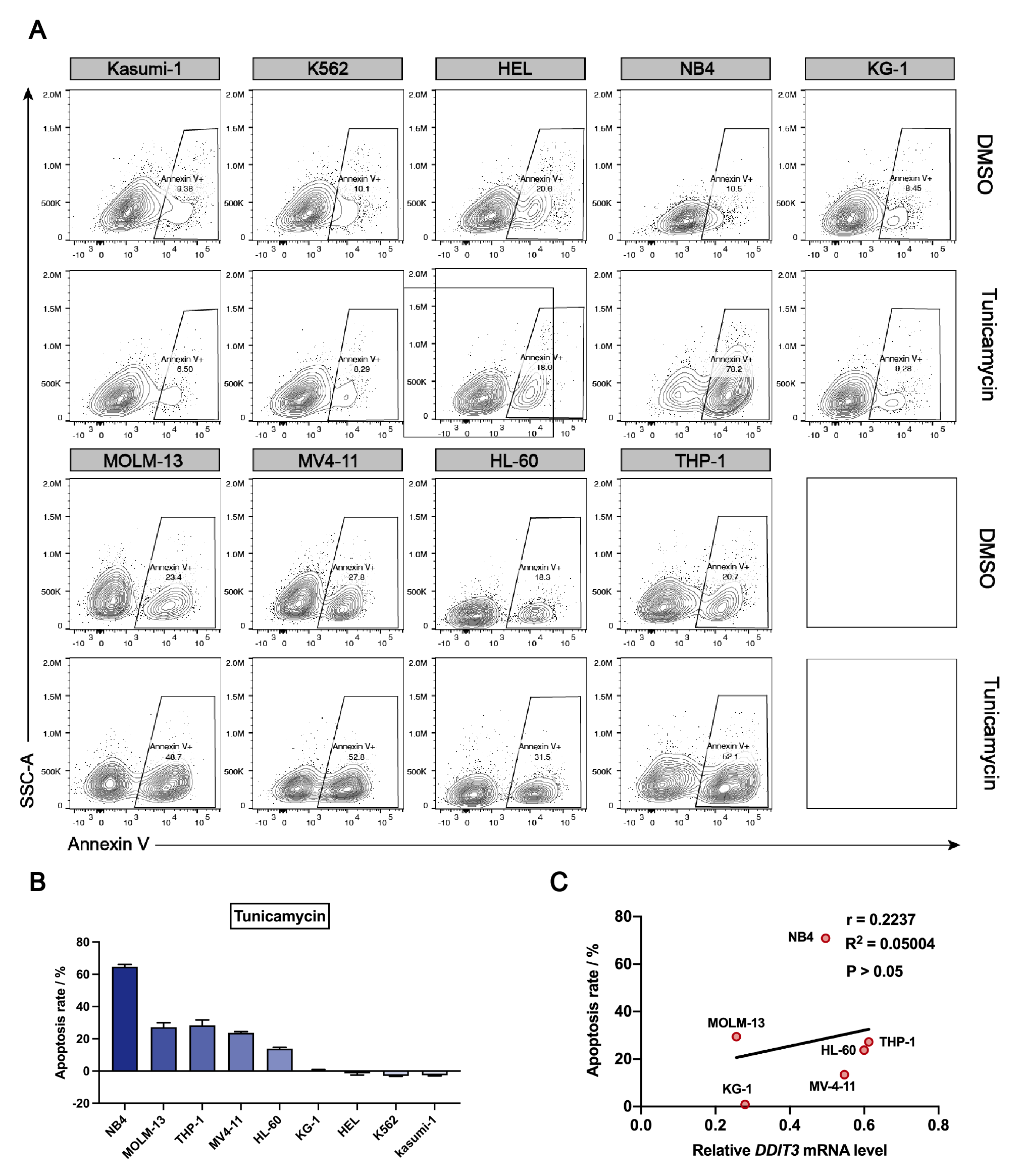


**Supplementary Fig. 2**

**Supplementary Fig. 3** Flow cytometric analysis revealed that compared with the negative control group, THP-1 cells with *CEBPA* knockdown displayed a significantly increased apoptosis rate after the treatment with tunicamycin (100 ng/ml, 48 hours).


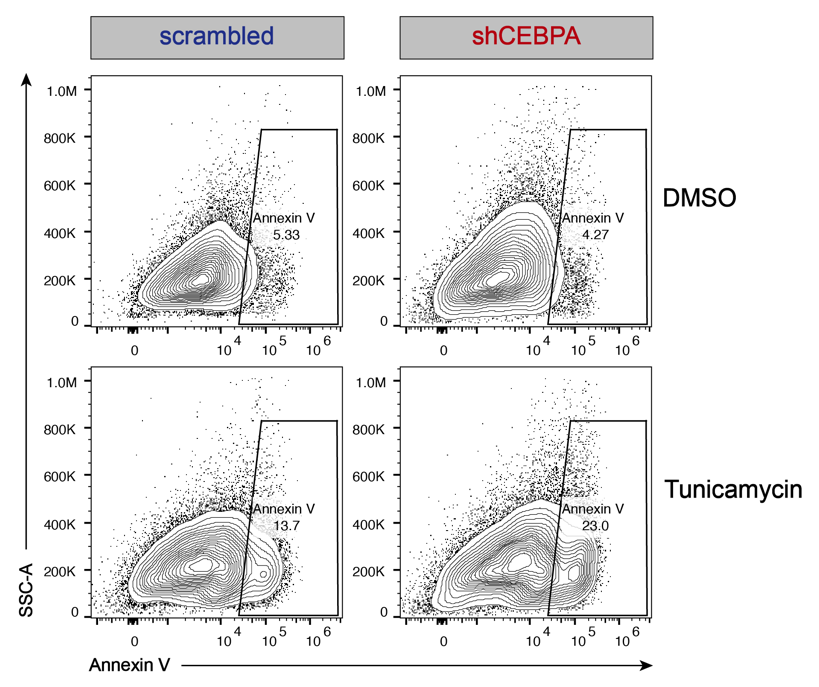


**Supplementary Fig. 3**

**Supplementary Fig. 4.** Flow cytometric analysis revealed that compared with the negative control group, 32Dcl3-shCebpa cells displayed a significantly increased apoptosis rate during granulocytic differentiation induced by G-CSF (100 ng/ml, 48 hours).


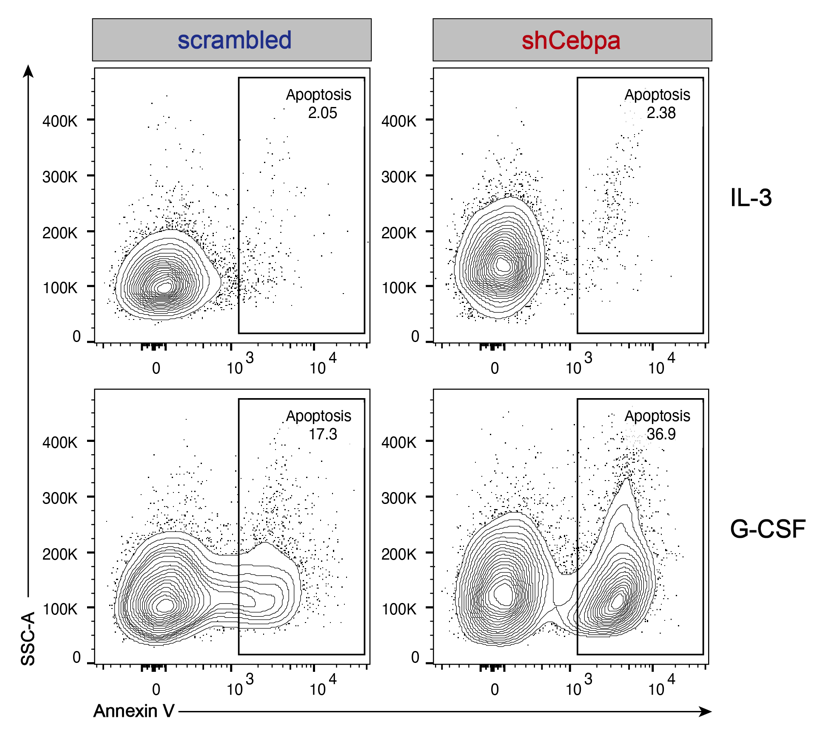


**Supplementary Fig. 4**

**Supplementary Fig. 5.** The C/EBPα p42/p30 ratio was calculated based on the relative optical density. The Pearson's correlation test was applied to determine the correlation of the C/EBPα p42/p30 ratio and DDIT3. A negative linear correlation trend was observed between the C/EBPα p42/p30 ratio and DDIT3 at the protein level.

**
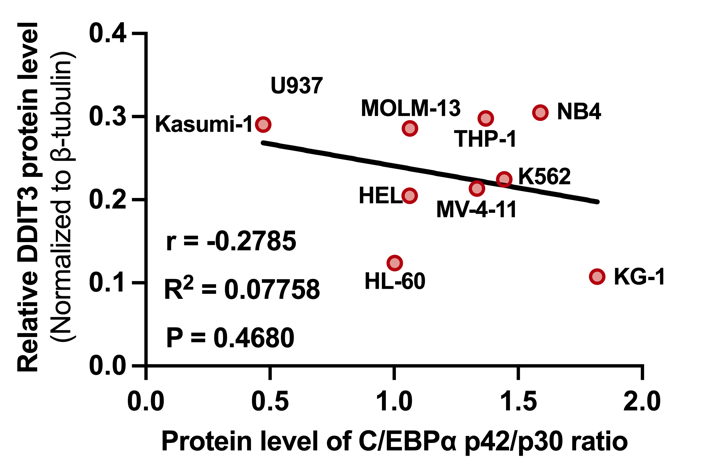
**

**Supplementary Fig. 5**

**Supplementary Fig. 6.** (A) Flow cytometric analysis revealed that compared with the negative control group, NB4 cells with the induction of C/EBPα-p42 displayed a significantly reduced apoptosis rate after the treatment with tunicamycin (100 ng/ml, 48 hours). (B) Flow cytometric analysis revealed that compared with the negative control group, THP-1 cells with the induction of C/EBPα-p30 displayed a significantly increased apoptosis rate after the treatment with tunicamycin (100 ng/ml, 48 hours).


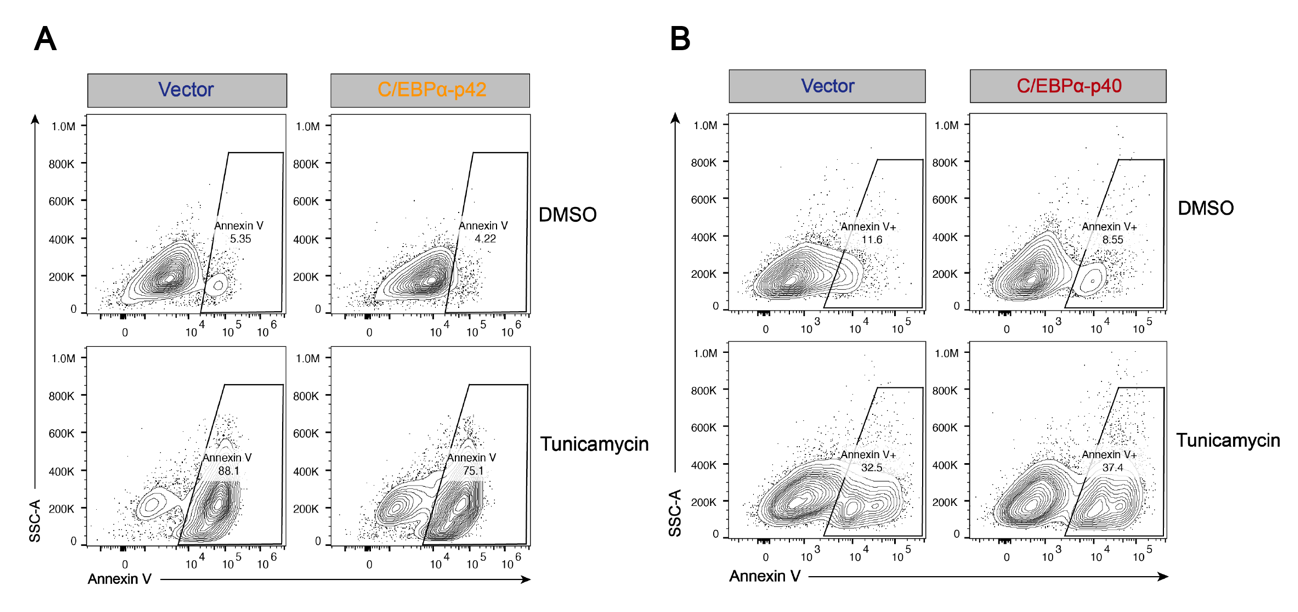


**Supplementary Fig. 6**

**Supplementary Fig. 7.** (A) Other four fragments in the promoter region of *DDIT3* were deleted (schematic diagram). (B) Deletions of other four fragments in the promoter region of *DDIT3* including Chr12:57521027-57521326, Chr12:57521327-57521626, Chr12:57521627-57522036 and Chr12:57522037-57522236 did not affect the function of both C/EBPα-p42 and C/EBPα-p30. (C) Schematic diagram of C/EBPα regulating DDIT3 and outcome of UPR. In *CEBPA* wild type AML cells, C/EBPα-p42 is the dominate isoform and competitively inhibits the function of C/EBPα-p30 to keep a low basal DDIT3 level. When faced with ER stress, cells tend to initiate adaptive UPR, leading to cell survival. As for low p42/p30 ratio AML cells, overexpressed C/EBPα-p30 isoform strongly increases the basal DDIT3 level. When faced with ER stress, cells tend to initiate terminal UPR, leading to cell apoptosis.

**
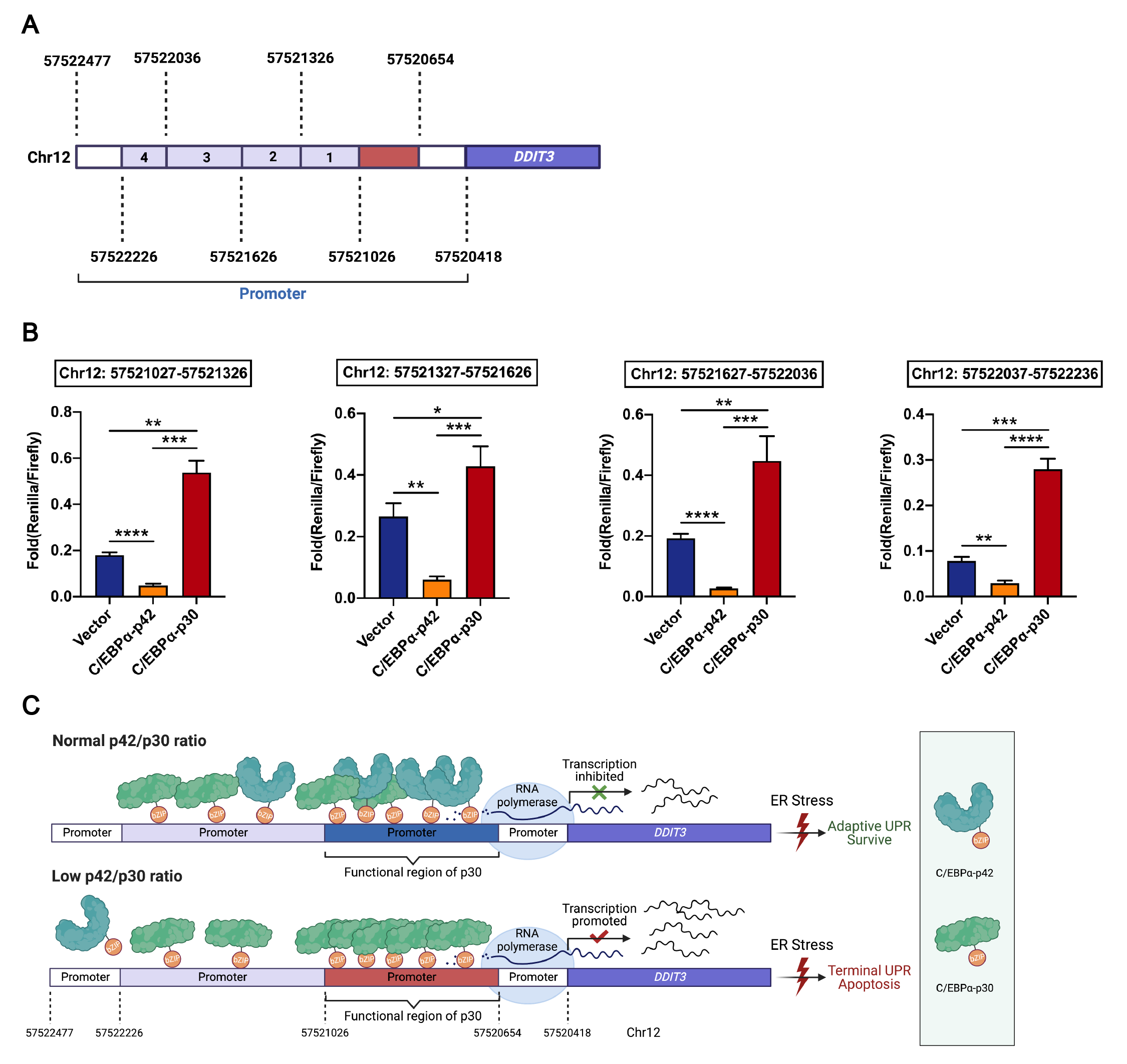
**

**Supplementary Fig. 7**

**Supplementary Fig. 8.** (A) The RNA-seq data from Beat AML project of AML patients was analyzed using cBioPortal database. A positive linear correlation was observed between *CEBPA* and *BCL2* expression in these cases, and negative linear correlations were observed between *CEBPA* and *BCL2L1* and *MCL1*. (B) The microarray data from GEO (GSE38987) (https://www.ncbi.nlm.nih.gov/gds) of AML patients was analyzed. A positive linear correlation was observed between *CEBPA* and *BCL2* expression in these cases, and negative linear correlations were observed between *CEBPA* and *BCL2L1* and *MCL1*. (C) The Pearson's correlation test was applied to determine the correlations between the protein levels of C/EBPα and BCL2, MCL1, and BCL-X_L_ in AML cell lines. A positive linear correlation was observed between C/EBPα and BCL2 expression, and negative linear correlations were observed between C/EBPα and BCL-X_L_ and MCL1. (D) The Pearson's correlation test was applied to determine the correlation between the C/EBPα p42/p30 ratio and BCL2, MCL1, and BCL-X_L_ in AML cell lines.


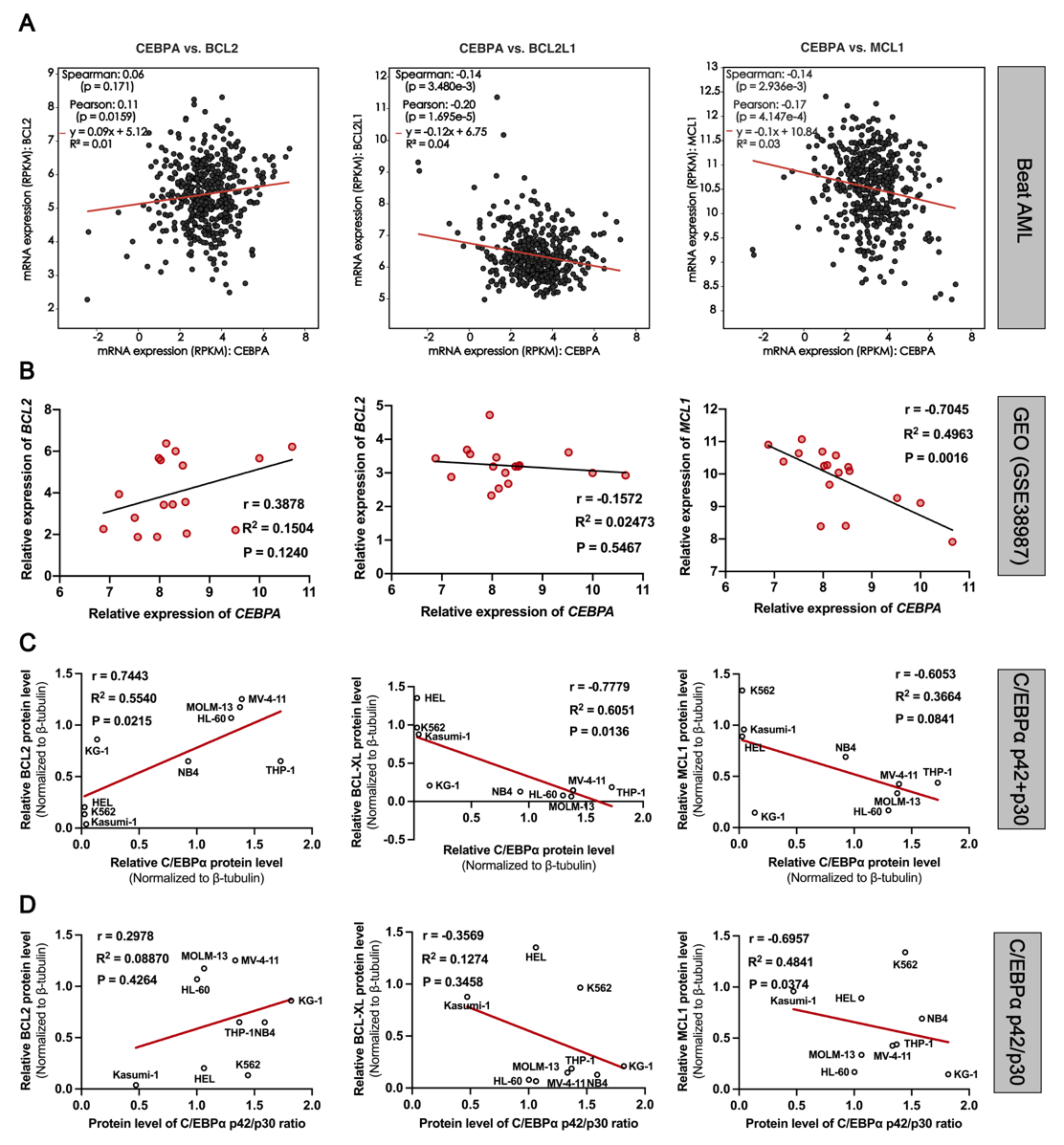


**Supplementary Fig. 8**

**Supplementary Fig. 9.** Flow cytometric analysis of the apoptosis rates of AML cell lines after the treatment of venetoclax (5 μM, 48 hours) and sorafenib (3 μM, 48 hours).


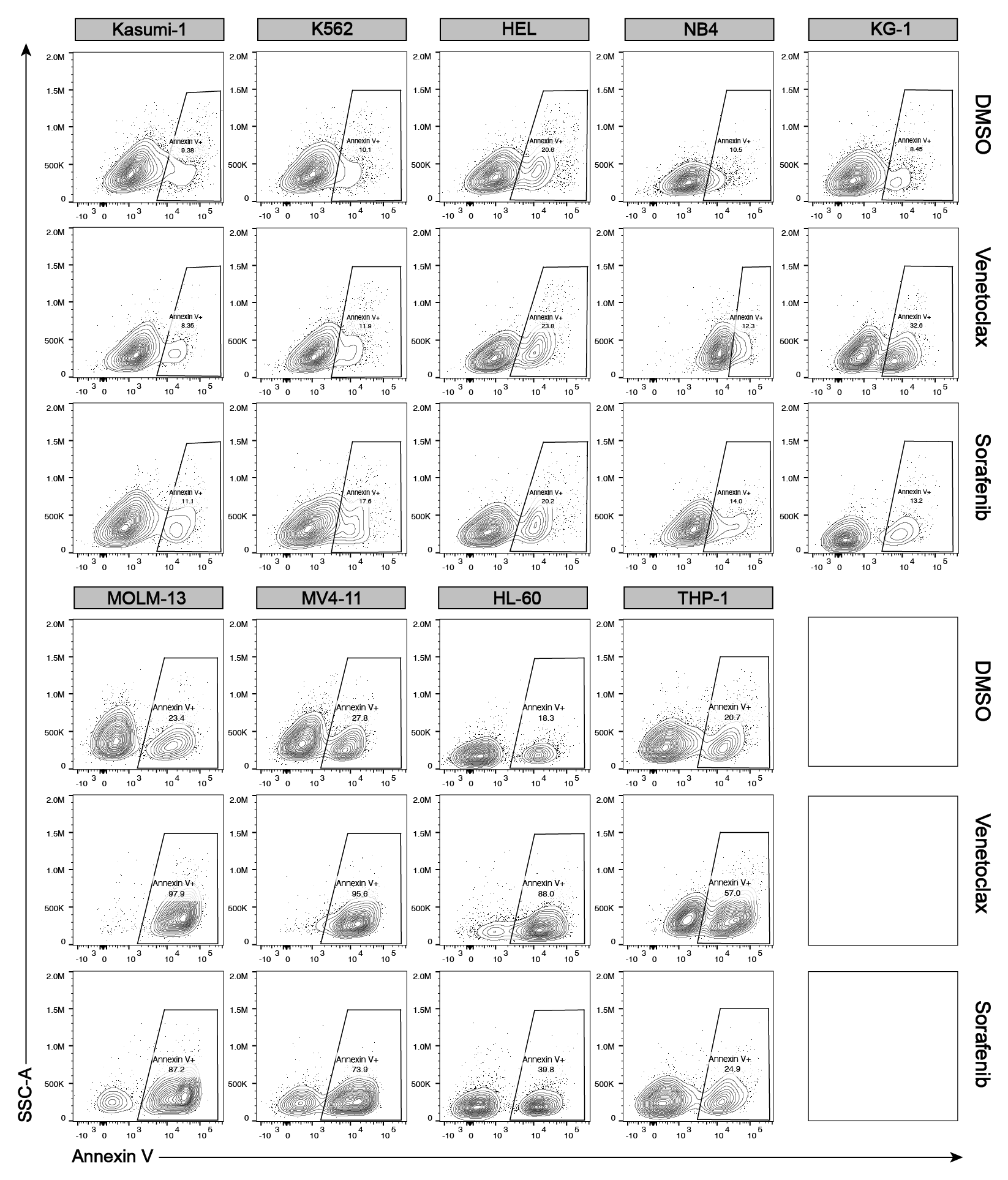


**Supplementary Fig. 9**

**Supplementary Fig. 10.** (A) The histogram of the apoptosis rates of AML cell lines after the treatment of venetoclax (5 μM, 48 hours) and sorafenib (3 μM, 48 hours). The results of Kasumi-1 were not shown because of a better survival rate was detected after venetoclax treatment. (B)-(C) The sensitivity to venetoclax cytotoxicity was positively relevant to the mRNA (B) and protein (C) levels of BCL2 in AML cell lines.


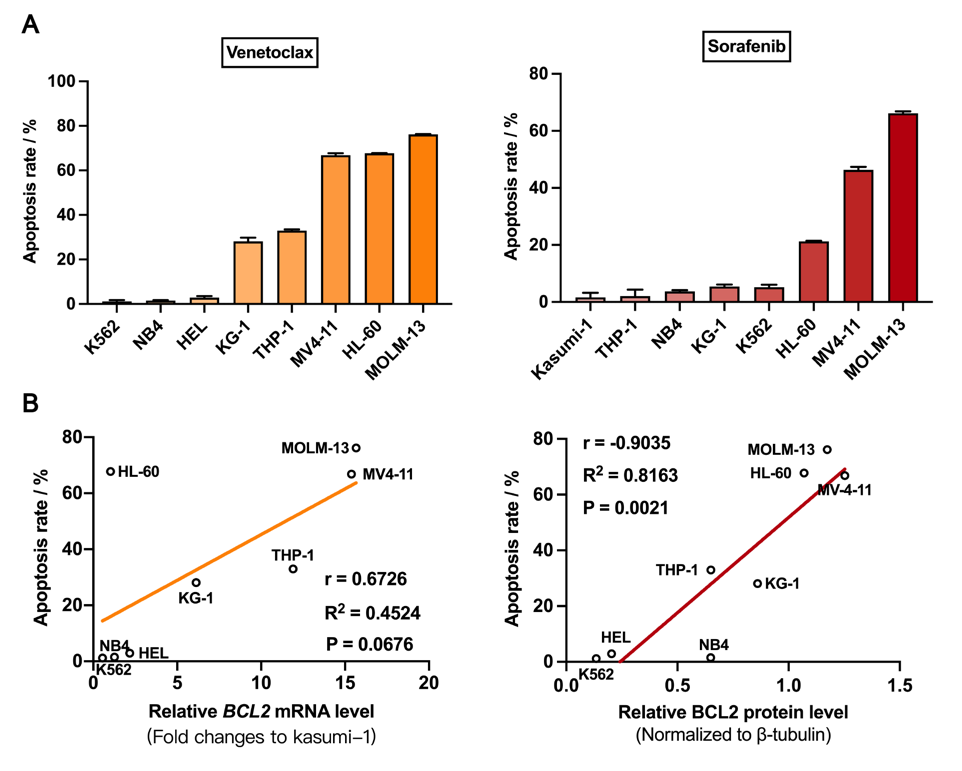


**Supplementary Fig. 10**

**Supplementary Fig. 11.** (A) Quantitative analysis of protein levels of BCL2, BCL-X_L_ and MCL1 in THP-1 cells with *CEBPA* knockdown. (B) Quantitative analysis of protein levels of BCL2, BCL-XL and MCL1 in NB4 cells with induction of C/EBPα-p42 and C/EBPα-p30. (C) Quantitative analysis of protein levels of BCL2, BCL-X_L_ and MCL1 in THP-1 cells with induction of C/EBPα-p30.


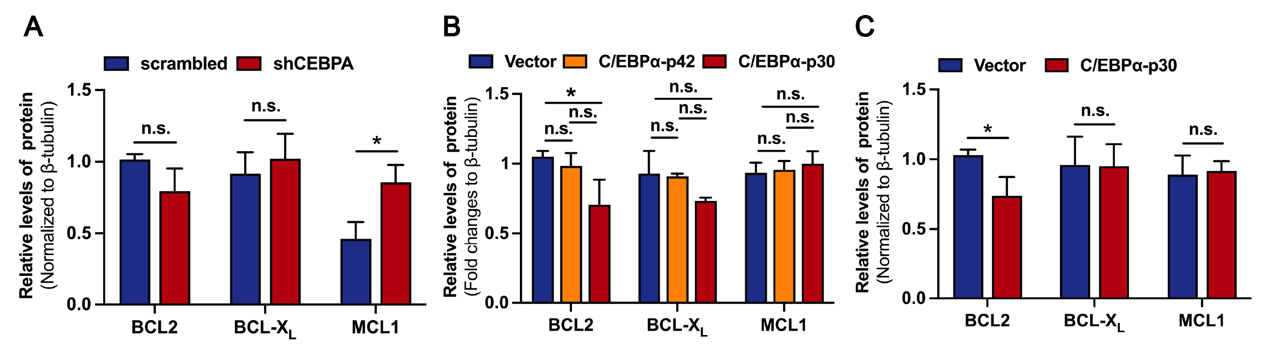


**Supplementary Fig. 11**

**Supplementary Fig. 12.** (A) The mRNA levels of DDIT3 were increased in NB4 cells after the treatment of tunicamycin (100 ng/ml, 24 hours) and sorafenib (3 μM, 24 hours). (B) Quantitative analysis of protein levels of DDIT3 in NB4 cells after the treatment of tunicamycin (100 ng/ml, 24 hours) and sorafenib (3 μM, 24 hours). (C) Correlation analysis using the Pearson model revealed that the mRNA levels of *CEBPA* in AML cell lines tended to be negatively correlated with tunicamycin (100 ng/ml, 48 hours) and sorafenib (3 μM, 48 hours) induced apoptosis rates.


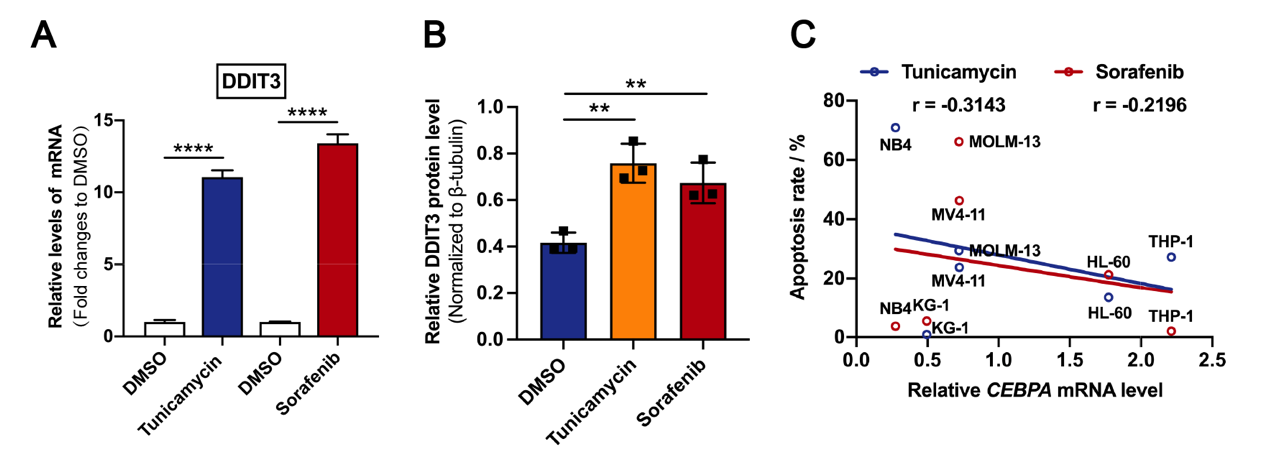


**Supplementary Fig. 12**

**Supplementary Fig. 13.** (A) Quantitative analysis of protein levels of BCL2, BCL-X_L_ and MCL1 in NB4 cells with combined treatment with tunicamycin and venetoclax and each drug alone. (B) Quantitative analysis of protein levels of BCL2, BCL-X_L_ and MCL1 in NB4 cells with combined treatment with sorafenib and venetoclax and each drug alone.


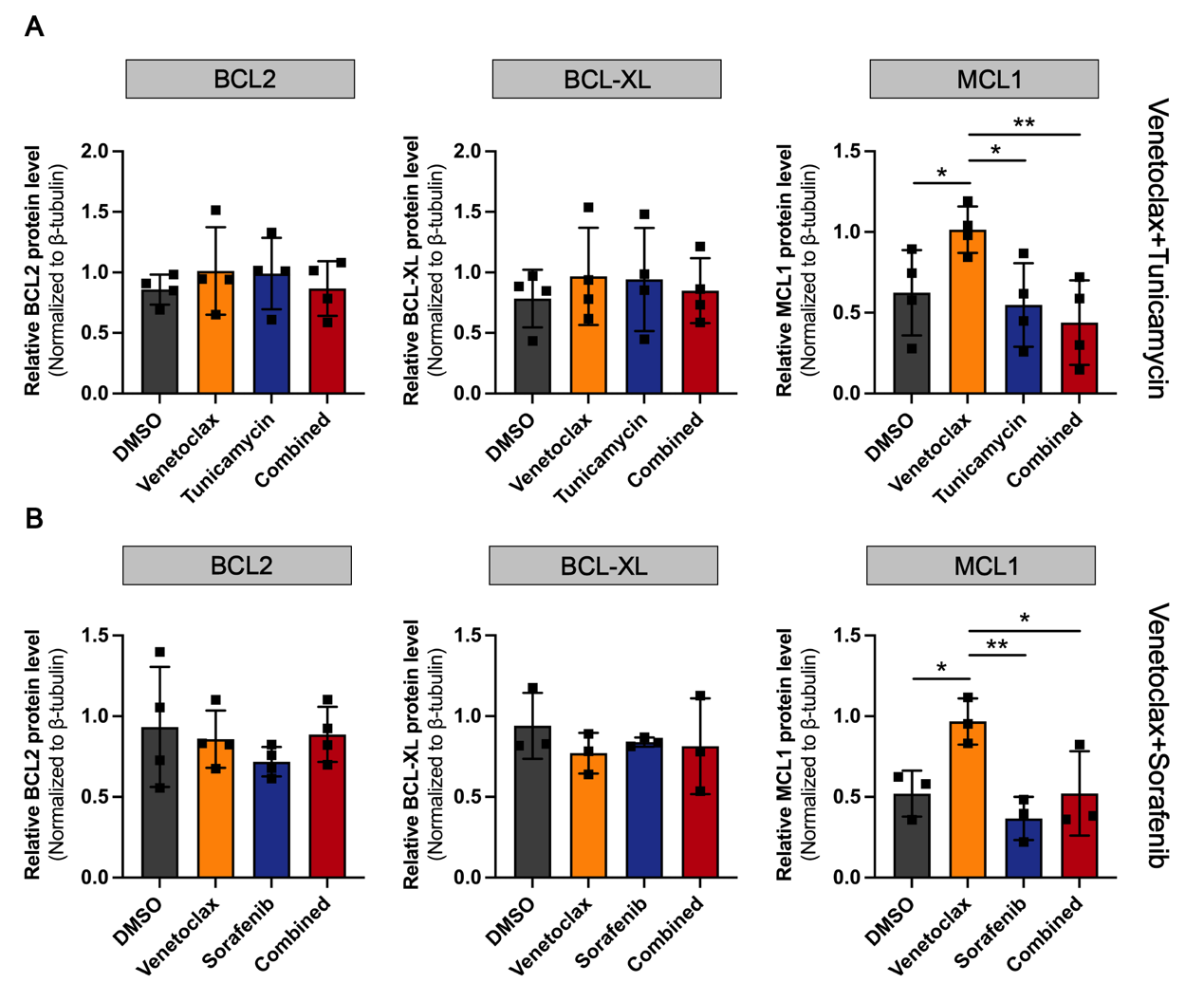


**Supplementary Fig. 13**

**Supplementary Fig. 14.** (A) The protein levels of p-ERK in NB4 cells were significantly downregulated by treatment with venetoclax+tunicamycin and venetoclax+sorafenib. (B) Quantitative analysis of protein levels of p-ERK and ERK after the treatment of DMSO, venetoclax, tunicamycin, venetoclax+tunicamycin, sorafenib and venetoclax+sorafenib.

**
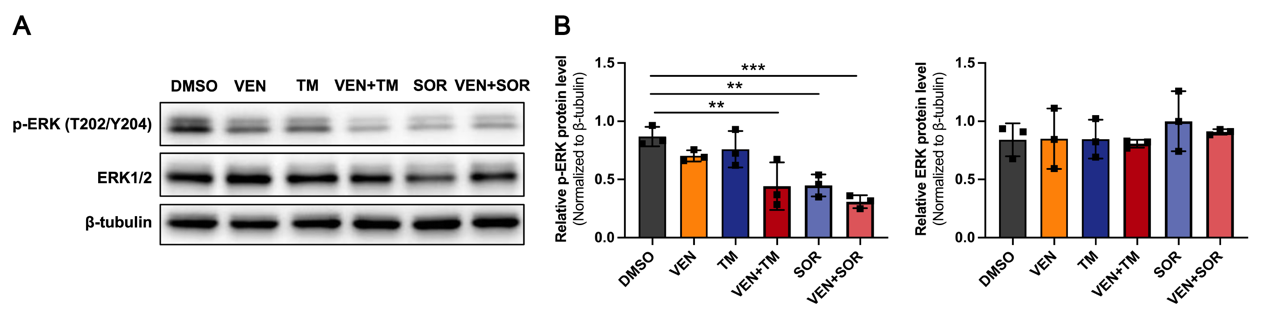
**

**Supplementary Fig. 14**

**Supplementary Fig. 15.** (A)-(B) Flow cytometric analysis revealed that combined treatment with tunicamycin (100 ng/ml) and venetoclax (5 μM) resulted in increased apoptosis/cell death of NB4 cells compared to either drug alone (A), and combined treatment with sorafenib (5 μM) and venetoclax (5 μM) also resulted in increased apoptosis/cell death compared to either drug alone (B).


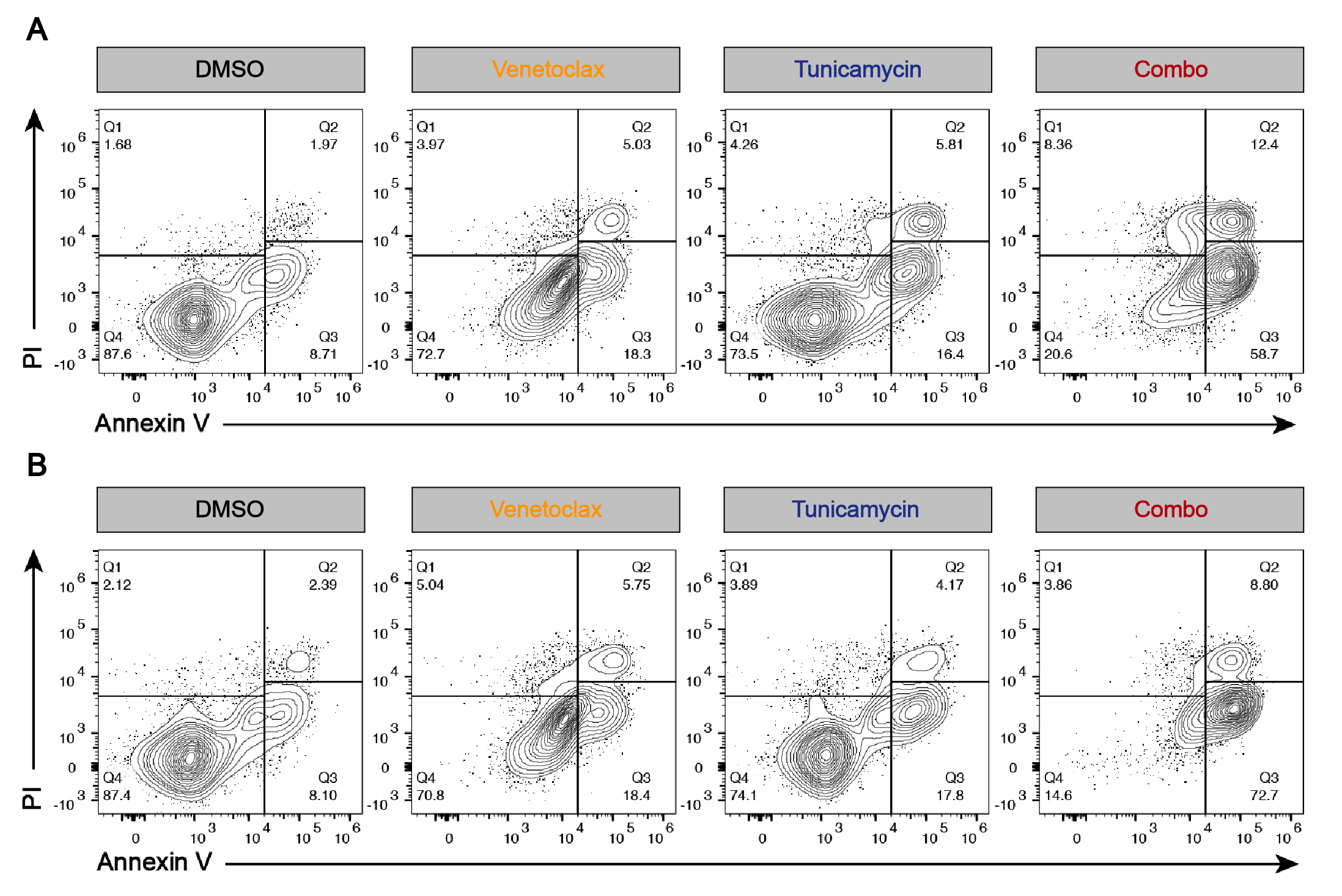


**Supplementary Fig. 15**

**Supplementary Fig. 16.** (A) BLI of leukemia growth on day 7 and the histogram. (B) BLI of leukemia growth on day 14 and the histogram.


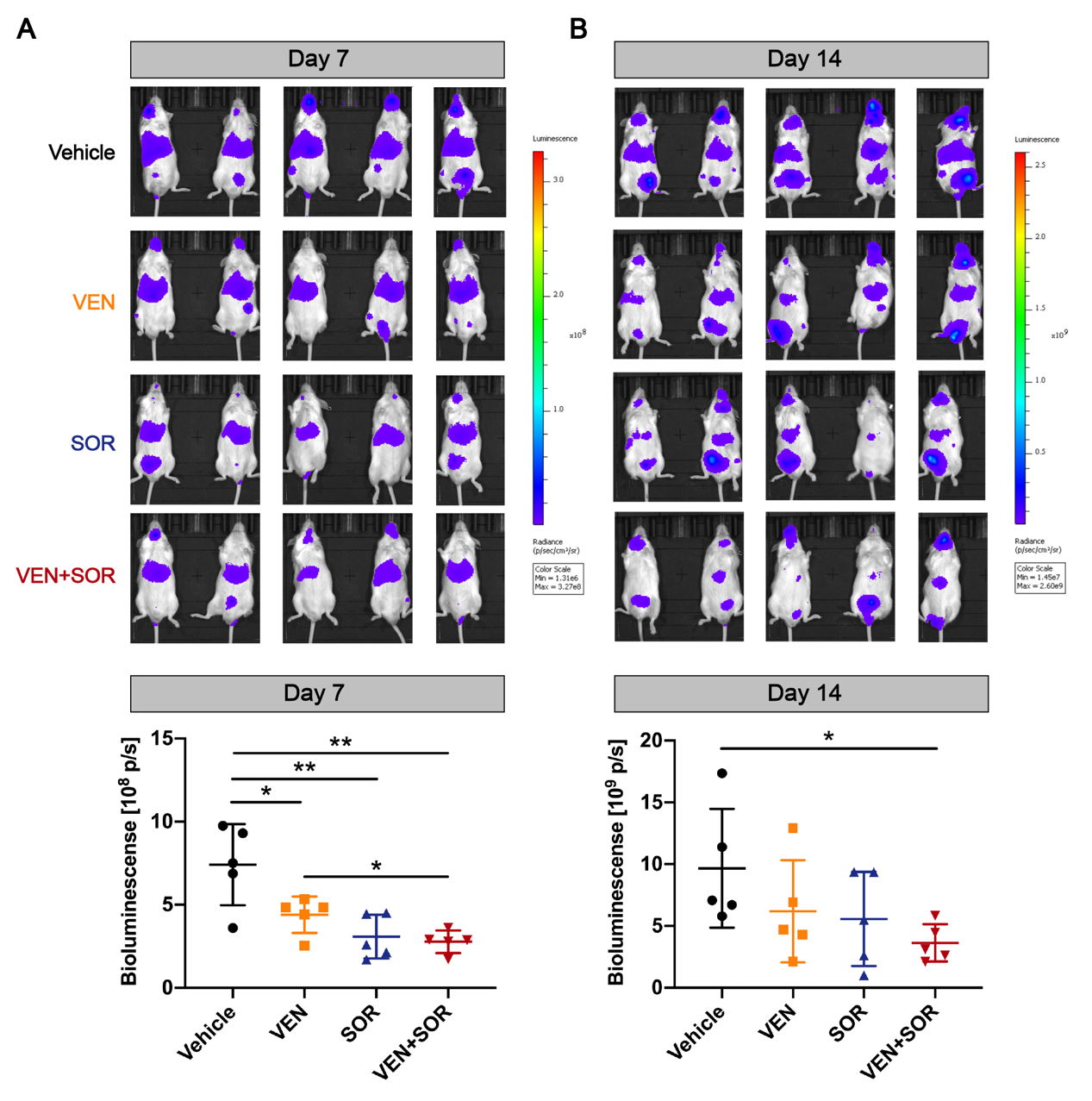


**Supplementary Fig. 16**
